# Projected effects of Climate‐change‐induced flow alterations on stream macroinvertebrate abundances

**DOI:** 10.1002/ece3.3907

**Published:** 2018-02-22

**Authors:** Karan Kakouei, Jens Kiesel, Sami Domisch, Katie S. Irving, Sonja C. Jähnig, Jochem Kail

**Affiliations:** ^1^ Department of Ecosystem Research Leibniz‐Institute of Freshwater Ecology and Inland Fisheries (IGB) Berlin Germany; ^2^ Department of Biology, Chemistry and Pharmacy Free University of Berlin Berlin Germany; ^3^ Department of Hydrology and Water Resources Management Institute for Natural Resource Conservation Christian‐Albrechts‐University Kiel Kiel Germany; ^4^ Department of Aquatic Ecology University of Duisburg‐Essen Essen Germany

**Keywords:** community responses, flow changes, flow preferences, global‐change effects, indicators of hydrologic alterations, species abundances, species responses

## Abstract

Global change has the potential to affect river flow conditions which are fundamental determinants of physical habitats. Predictions of the effects of flow alterations on aquatic biota have mostly been assessed based on species ecological traits (e.g., current preferences), which are difficult to link to quantitative discharge data. Alternatively, we used empirically derived predictive relationships for species’ response to flow to assess the effect of flow alterations due to climate change in two contrasting central European river catchments. Predictive relationships were set up for 294 individual species based on (1) abundance data from 223 sampling sites in the Kinzig lower‐mountainous catchment and 67 sites in the Treene lowland catchment, and (2) flow conditions at these sites described by five flow metrics quantifying the duration, frequency, magnitude, timing and rate of flow events using present‐day gauging data. Species’ abundances were predicted for three periods: (1) baseline (1998–2017), (2) horizon 2050 (2046–2065) and (3) horizon 2090 (2080–2099) based on these empirical relationships and using high‐resolution modeled discharge data for the present and future climate conditions. We compared the differences in predicted abundances among periods for individual species at each site, where the percent change served as a proxy to assess the potential species responses to flow alterations. Climate change was predicted to most strongly affect the low‐flow conditions, leading to decreased abundances of species up to −42%. Finally combining the response of all species over all metrics indicated increasing overall species assemblage responses in 98% of the studied river reaches in both projected horizons and were significantly larger in the lower‐mountainous Kinzig compared to the lowland Treene catchment. Such quantitative analyses of freshwater taxa responses to flow alterations provide valuable tools for predicting potential climate‐change impacts on species abundances and can be applied to any stressor, species, or region.

## 1 INTRODUCTION

River biota depend on a range of environmental variables, including natural habitat conditions as well as stressors. While the effects of a variety of environmental variables and stressors such as land‐use, climate, and substrate conditions on riverine species are well understood (Miserendino et al., 2011 *;* Schröder et al., 2013), the relationship between riverine species’ abundances and river flow is less often explored (Kuemmerlen et al., 2014, 2015; Pyne & Poff, 2017), although it has been widely stated that flow (i.e., discharge) is one of the key habitat variables in river ecosystems (Arthington, Bunn, Poff, & Naiman, 2006; Dewson, James, & Death, 2007; Domisch et al., 2017; Poff et al., 1997).

Flow alterations are among the most important stressors that affect river habitats (Vörösmarty et al., 2010), and different organism groups strongly respond to flow alterations (Bunn & Arthington, 2002; Kuemmerlen et al., 2015; Lloyd et al., 2003; Lytle, Merritt, Tonkin, Olden, & Reynolds, 2017; Poff & Zimmerman, 2010; Pyne & Poff, 2017; White et al., 2017). Regional precipitation patterns and variability are likely to change until mid‐century, for example, increasing number of extreme events (Nilson & Krahe, 2014). As river flow conditions are precipitation‐driven, they may respond directly to climate change (Filipe et al., 2013; Wenger et al., 2011; Woodward, Perkins, & Brown, 2010), and severe flow alterations are to be expected.

Several studies have already assessed the ecological response of stream macroinvertebrates to climate change (Poff & Zimmerman, 2010 and references therein; Floury, Usseglio‐Polatera, Ferreol, Delattre, & Souchon, 2013; Chessman, 2015). In the absence of long‐term observational data, they focused on species ecological traits as the basis for their analyses. Species ecological traits have been reported to be informative and best‐case data for providing clues to the poorly understood mechanisms that threaten species occurrences in their environment (Matthews & Marsh‐Matthews, 2003). Moreover, potential responses and range shifts of species to climate‐change impacts might be identified by their ecological traits (Hamilton, Stamp, & Bierwagen, 2010). For example, a strong correlation between medium‐/high‐flow conditions and the occurrence of rheophilic species suggests that a projected decrease in flow conditions may have a major impact on the occurrence of these species (e.g., Chessman, 2015; Thomson et al., 2012). However, as traits information are often qualitative data stemming from literature reviews and expert knowledge (Schmidt‐Kloiber & Hering, 2015), it is difficult to link traits to quantitative data and they are less suited to quantitatively assess and predict the effects of flow changes (e.g., discharge changes due to climate change).

Only recently, discharge data have been used to empirically derive quantitative flow preferences for macroinvertebrates (Kakouei, Kiesel, Kail, Pusch, & Jähnig, 2017). These flow preferences reveal species response (SR) along the range of flow conditions. The information on flow conditions is described by key flow metrics, for example, the indicators of hydrologic alterations—also known as IHA metrics (Richter, Baumgartner, Powell, & Braun, 1996). The IHA metrics provide information on the duration, magnitude, frequency, timing, and rate of flow events for present patterns and also for potential future changes. The effects of climate change on ecologically important attributes of flow conditions (e.g., extreme events) have the potential to threaten ecosystem functioning (Jentsch & Beierkuhnlein, 2008) by causing ecological changes in the structure and composition of aquatic communities (Poff & Zimmerman, 2010; Pyne & Poff, 2017).

Here, we introduce an approach that can be used to quantitatively predict the impacts of climate change‐induced flow alterations on the abundance of stream macroinvertebrates. We compared the predicted species’ abundances in two contrasting catchments differing in flow regime and species pool to answer the following questions:

1. In which regard do the climate change‐induced changes in discharge (different flow conditions according to IHA metrics) have varying effects on stream macroinvertebrates’ abundances? And changes in which flow metrics will potentially have the largest impact?
2. How do possible climate‐change impacts on species’ abundances, mediated through flow, differ between the two catchments?

## 2 METHODS

### 2.1 Study area

**Figure 1:**
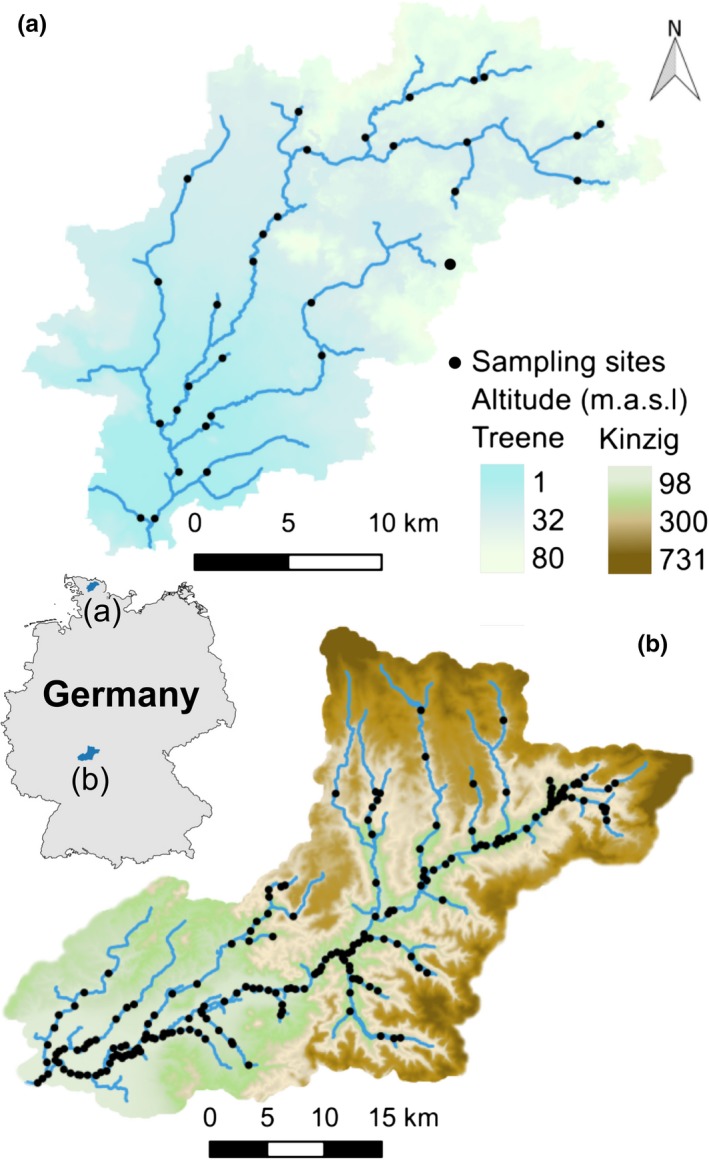
The study area: the Treene catchment in lowland (a) and the Kinzig catchment in the lower‐mountainous region (b) in Germany

The effect of climate change‐induced flow alterations on river macroinvertebrates was assessed in two case‐study catchments in Germany to investigate potential differences between effects in different ecoregions: the central lower‐mountainous region (Kinzig catchment) and the northern lowlands (Treene catchment, Figure 1, Table 1).

**Table 1:** Catchment characteristics of the two study catchments

Catchment characteristic	Treene	Kinzig
River basin	Eider	Main
Ecoregion	Lowland	Lower‐mountain region
Number of river orders	3	3
Catchment size at outlet [km^2^]	481	1,175
Elevation gradient [m a.s.l.]	1–80	98–731
Major land‐use classes	Agriculture (48%) Pasture (32%)	Forest (45%) Pasture (22%)
Mean annual precipitation [mm]	887	859
Mean runoff rate (L s^−1^ km^−2^]	13.2	10.7
Mean discharge [m^3^/s]	6.23	10.48
Maximum discharge [m^3^/s]	34.9	165
Mean channel slope [%]	1.29	10.37
Median slope [%]	0.93	8.23

The following datasets were gathered in each catchment: (1) stream macroinvertebrate samples, (2) temporally corresponding gauge data for calibrating hydrological models and setting up predictive relationships between macroinvertebrates and flow conditions (i.e., discharge), and (3) projected high‐resolution climate model data for simulating projected changes in flow conditions and deriving changes in SR.

### 2.2 Biological data

For both river catchments, macroinvertebrate sample data were gathered from regional authorities. Samples were taken between 2005 and 2012 in the Kinzig catchment and between 2004 and 2015 in the Treene catchment. Sampling and identification was carried out according to the standardized multihabitat sampling protocol (Haase et al., 2004), where each sample is representative of a 100‐m river reach. All taxa were identified to the species level. The datasets consisted of 225 samples from 176 sites in the Kinzig and 70 samples from 30 sites in the Treene catchment (Figure 1). Species occurring at less than eight sampling sites were excluded, as these data might affect the robustness of the statistical analyses (Heino & Soininen, 2010; Leigh & Datry, 2016), which reduced the number of modeled species from 150 to 134 in the Kinzig and from 78 to 60 in the Treene catchment (Table S1).

### 2.3 Flow data

Catchment borders and river networks used in this study were obtained from a digital elevation model with a 25‐m resolution (Hessian Administration for Soil Management and Geo‐information, and the Land Survey office Kiel). The obtained river network had 14,067 and 5,863 grid cells for the Kinzig and the Treene, respectively. All geoprocessing procedures were carried out using the open‐source software QGIS (QGIS Development Team, 2017).

To obtain flow data for each grid cell along the river network, the daily discharge time series (m^3^/s) from six (Kinzig) and four (Treene) gauging stations were extrapolated. Flow accumulation values were calculated for all sites/grid cells, providing the number of upstream cells that flow into that site/grid cell, ${\text{FA}}_{s_{i}}$. This drainage area of the site/grid cell was then related to the drainage area of the nearest gauging station, FA_g_, and the flow accumulation approach was used to calculate the mean daily discharge at all sites/grid cells along the river network, ${\text{MDD}}_{s_{i}}$, based on the mean daily discharge at the gauge MDD_g_:

(1) $${\text{MDD}}_{s_{i}}=\left(\frac{{\text{MDD}}_{g}}{{\text{FA}}_{g}}\right).\;{\text{FA}}_{s_{i}}$$

To obtain future projections of discharge, the hydrological processes in both catchments were modeled by the ecohydrological model SWAT (Soil and Water Assessment Tool; Arnold, Srinivasan, Muttiah, & Williams, 1998). SWAT is a semi‐distributed ecohydrological model that is used to calculate river discharge based on physical catchment data and climate time series. SWAT delineates a given catchment into sub‐basins, which are further divided into areas with similar soil, land‐use, and slope (i.e., hydrologic response units, HRUs). Processes such as evapotranspiration, surface runoff, interflow and groundwater components, infiltration, and soil water storage are depicted in each HRU and then aggregated to the sub‐basin scale (Guse et al., 2015). This procedure led to 22 sub‐basins in the Kinzig and 13 sub‐basins in the Treene catchment, for which daily simulated discharge data were available. The historical period from 1997 to 2015 was used to calibrate and validate the models. IHA metrics were calculated from simulated and observed discharge and the difference between the simulated and observed IHA metrics minimized during the calibration process (Kiesel et al., 2017).

Climate change data for SWAT were prepared from the CORDEX (Jacob et al., 2014) daily precipitation and minimum and maximum temperature dataset for Europe for the RCP 8.5 scenario. We selected this scenario because it is considered the worst‐case scenario and represents the most severe conditions, meaning that this scenario would set the upper limit for potential taxa responses. The CORDEX dataset provides the most recent and most detailed (11‐km resolution) climate change dataset for Europe. All 16 available global climate models and regional climate models were downloaded (ESGF, 2016), and the time series were extracted from all climate stations where observed data were available for bias correction. The time series were bias‐corrected using six methods (linear scaling, delta change, distribution mapping, local intensity scaling, and power transformation; Teutschbein & Seibert, 2012). All combinations of model types and bias corrections (in total, 80 per catchment) were run in the calibrated SWAT models for the Kinzig and Treene catchments (unpublished data). The hindcasted climate data from the global climate model MOHC‐HadGEM2‐ES, combined with the regional climate model CLMcom‐CCLM4‐8‐17 and the bias correction method “distribution mapping,” performed best in depicting the historic flow conditions in the Treene and Kinzig catchments; hence, this was the method also used for climate change predictions in this study. The CORDEX data were used for both the baseline (hindcasted) and the future conditions to ensure that results were not affected by differences between modeled and observed climate data.

### 2.4 Preselection and calculation of IHA metrics

The 177 IHA metrics (Olden & Poff, 2003) were grouped into five categories that provide information on changes in duration, magnitude, frequency, timing, and rate of flow events. All 177 IHA metrics were calculated for all sampling sites according to the flow data 12 months before the biological sampling using the flow data from the historical period 1997–2015 for each SWAT sub‐basin. To avoid redundancy, one metric per IHA category was selected in each river catchment according to the following criteria: (1) The pairwise correlation between IHA metrics should not exceed the sensitivity threshold of |*r*| > .7 (Dormann et al., 2013), and (2) if it exceeds this threshold, the metric with the lower loading on the most significant principal component axes was excluded (for details see Olden and Poff (2003) and Kakouei et al. (2017)).

The criteria resulted in the selection of different IHA metrics in the two study catchments (Table 2) due to differences in the flow regime and climatic‐/hydro‐morphological conditions in lower‐mountainous versus lowland regions. Some metrics were highly cocorrelated in the lowland Treene, while pairwise correlations remained below the sensitivity threshold in the lower‐mountainous Kinzig catchment. However, the selected metrics covered all five IHA categories; therefore, a diverse range of possible environmental responses to climate change‐induced flow alterations was expected (Burn & Soulis, 1992). All other metrics were cocorrelated (|*r*| > .7) with at least one of the selected metrics in this study.

**Table 2:** Descriptions, calculation procedures, units, and temporal aspects of the five IHA metrics used in Treene and Kinzig catchment, respectively; one IHA metric per category (according to Olden & Poff, 2003 and references therein)

Catchment	IHA metric (code, category)	Description	Calculation procedure	Unit	Temporal aspect
Treene	Duration of high‐flow events (dh4, duration)	Annual maximum 30‐day moving average flows	Compute the max of 30‐day moving average flows	m^3^/s	Daily
	Frequency of low‐flow events (fl2, frequency)	Variability in low pulse count	fl1 computes the average number of flow events with flows below a threshold equal to the 25th percentile value for the entire flow record. To compute fl2, the standard deviation in the annual pulse counts was calculated for fl1, and fl2 is 100 times the standard deviation divided by the mean pulse count	%	Annual
	Magnitude of low‐flow events (ml16, magnitude)	Median of annual minimum flows	Compute the median of the ratios of minimum annual flows to the median flow for each year	Dimensionless	Interannual
	Rate of change in flow events (ra7, Rate)	Negative change of flow	Compute the change in log of flow for days in which the change is negative for the entire flow record	m^3^/s	Daily
	Timing of high‐flow events (ta3, timing)	Seasonal predictability of flooding	Divide the period up into 2‐month periods (i.e., October–November, December–January, etc.). Count the number of flood days (flow events with flows >1.67‐year flood) in each period over the entire flow record. ta3 is the maximum number of flood days in any one period divided by the total number of flood days	Dimensionless	Annual
Kinzig	Duration of high‐flow events (dh4, duration)	Annual maximum 30‐day moving average flows	Compute the max of 30‐day moving average flows	m^3^/s	Daily
	Frequency of low‐flow events (fl1, frequency)	Low‐flow pulse count	Compute the average number of flow events with flows below a threshold equal to the 25th percentile value for the entire flow record	Number of events/year	Annual
	Magnitude of low‐flow events (ml18, magnitude)	Variability in base‐flow index	Compute the standard deviation for the ratios of minimum 7‐day moving average flows to mean annual flows for each year	%	Annual
	Rate of change in flow events (ra4, Rate)	Variability in fall rate	Compute the standard deviation for the negative flow changes	%	Annual
	Timing of low‐flow events (th3, timing)	Seasonal predictability of nonflooding	Computed as the maximum proportion of a 365‐day year that the flow is less than the 1.67‐year flood threshold. Accumulate nonflood days that span all years. The th3 is maximum length of those flood‐free periods divided by 365	Dimensionless	Annual

**Figure 2:**
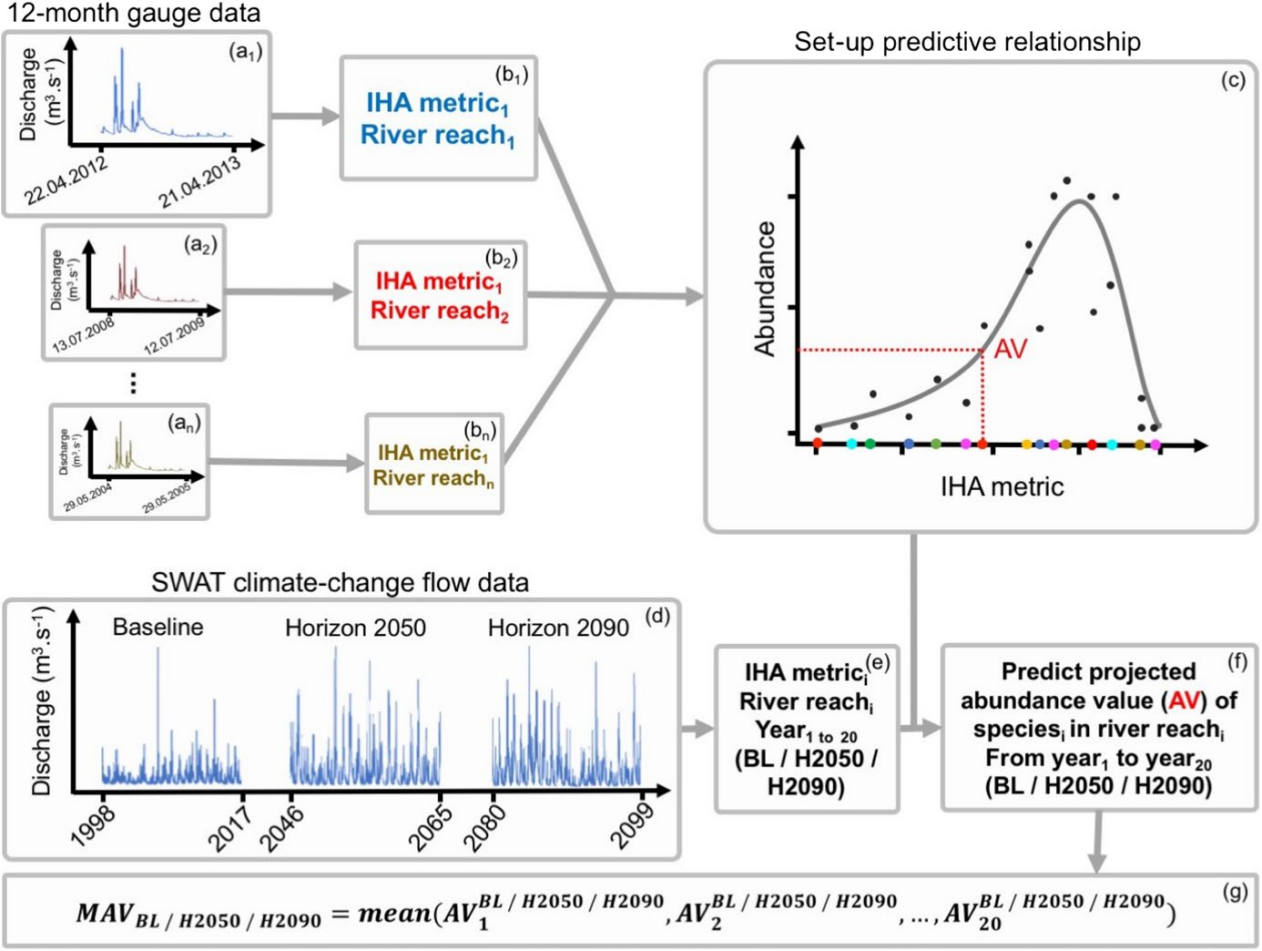
Workflow schematic of the analyses for one species and one IHA metric. The predictive relationship (c) was set up by calculating each IHA metric for each sample (b) using the 12‐month time‐series gauge data before the date of biological sampling (a). Each IHA metric (e) was then calculated for each year during baseline (BL, 1998 ‐ 2017, d), horizon 2050 (H2050, 2046 ‐ 2065, d), and horizon 2090 (H2090, 2080 ‐ 2099, d) and then used to predict projected abundance values (AV, f) for each species in each year during each period. The 20 AV per species were averaged to calculate the mean abundance value (MAV, g) for each species in each period

For all sampling sites in both river catchments, the IHA metrics (Figure 2b) were calculated based on the extrapolated gauge data from the 12‐month period prior to the date of the biological sampling (Figure 2a). This period is expected to represent the effects of flow conditions on macroinvertebrates for a sample (Jourdan et al., 2018; Leigh & Datry, 2016). For example, for a macroinvertebrate sample from 21.04.2013, flow data between 22.04.2012 and 21.04.2013 were considered.

### 2.5 Temporal pseudo‐replication

Some samples were taken at the same sampling site but at different dates. To avoid temporal pseudo‐replication (Hale, Noble, Piper, Garmire, & Tonsor, 2016; Hurlbert, 1984), only biological samples taken at the same sampling site but sampled at least 12 months apart were considered as temporally independent and were included in the analysis (Kakouei et al., 2017). The 12‐month time period did overlap for two (Kinzig) and three (Treene) samples taken at the same site, slightly lowering the number of samples from 225 to 223 in the Kinzig and from 70 to 67 in the Treene, respectively.

### 2.6 Set‐up of predictive relationships

The predictive relationships were derived using hierarchical logistic regression modeling (Huisman, Olff, & Fresco, 1993; Jansen & Oksanen, 2013). SRs to each of the five IHA metrics were tested by seven logistic regression models with hierarchically increasing complexity (for details, see Jansen and Oksanen, 2013 and Kakouei et al., 2017), including all five Huisman–Olff–Fresco models and two extended models: the eHOF models flat response (I), monotone in‐/decreasing (II), interval optimum (III), symmetrical (IV), skewed (V), and the two extended models bimodal response with equal optima (VI), and bimodal response with unequal optima (VII). The ability of each model to support the data and to fit the observations was evaluated by comparing the Akaike information criterion (AICc).

For each taxon, the model explaining best its abundance using the specific IHA metric (Figure 2b) was then used as the predictive relationship for that IHA metric (Figure 2c).

#### 2.6.1 Predictive ability of best selected eHOF models

For each taxon, the predictive ability of the best model for each of the five IHA metrics was quantified by randomly separating the presences (observations, i.e., abundance data) and absences into training (75% of presences and 75% of absences) and testing (25% of presences and 25% of absences) datasets. We ran this random selection process 100 times, calculated the area under the receiver operating characteristic curve (AUC) for the test dataset, and subsequently averaged the 100 AUC scores per species (see Table S1 of the supplementary material for all model scores). The AUC measures the model's ability to discriminate between true and false positives (Hosmer, Lemeshow, & Sturdivant, 2013). AUC values range from 0.5 (model is no better than random) to 1 (perfect discrimination). Hosmer et al. (2013) report that AUC values <0.7 represents a sensitive threshold of adequate model discrimination, a score that was not met by 17 species regarding the timing of high‐flow events (ta3) in the Treene catchment. We decided to keep all species in our analyses, but accounted for the model skill via a weighting scheme that was proportional to the model skill (the better the AUC, the higher the influence of the species in the final species assemblage response analysis). We used a continuous weighting factor from one to two with 0.02 intervals.

The AUC values were calculated using the “multiclass.ROC” function in the R‐package “pROC,” which builds multiple receiver operating characteristic (ROC) curves to compute the multiclass AUC (Robin et al., 2011).

#### 2.6.2 Other environmental variables

The in situ occurrence and ecological response of stream macroinvertebrates depend on a variety of environmental variables, for example, land‐use, precipitation, and temperature (Pyne & Poff, 2017; Stoll, Breyer, Tonkin, Früh, & Haase, 2016; Tonkin, Stoll, Jähnig, & Haase, 2016). Precipitation is highly cocorrelated with discharge. Although none of these variables were directly used as covariates in this analysis, several variables (e.g., soil, land‐use and management, elevation and slope, precipitation, temperature, wind, humidity, and solar radiation) were considered in the SWAT hydrological models and, hence, were not duplicated as direct covariates in the modeling of taxa responses to flow alterations.

### 2.7 Potential responses of individual species and assemblages of river reaches

To account for the natural annual precipitation and discharge fluctuations (i.e., differences between wet and dry years), we compared three 20‐year periods instead of single years: a baseline period (i.e., current flow conditions from 1998 to 2017) and two future projected periods (named here as “horizon 2050” for the period between 2046 and 2064, and “horizon 2090” from 2080 to 2099; Figure 2d). For each biological sampling site, the flow data modeled by SWAT (Figure 2d) were used to compute a single IHA‐metric value (Figure 2e) and to predict species abundance values (AV, Figure 2f) for each year (12‐month period) of the three 20‐year periods, resulting in 60 IHA‐metric values and abundance values per species. The 20 AV per species were used to calculate a mean abundance value (MAV) for each of the three 20‐year periods (MAV_baseline_, MAV_horizon 2050_, MAV_horizon 2090_, Figure 2g).

The ratio between the mean response value of the baseline and the two future time periods was used to assess the effect of changes in each IHA metric (*m*_*i*_) on each species (*sp*_*i*_) at each sampling site (*s*_1_) by calculating percent change (∆‐Response):

(2) $$\left\{\left\{\Delta R_{\mathrm{horizon}\;2050}^{m_{i}}=\frac{{\text{MAV}}_{\mathrm{horizon}\;2050}^{m_{i}}.100}{{\text{MAV}}_{\mathrm{baseline}}^{m_{i}}}\right\}\begin{array}{c} \;sp_{n}\;\\ \;\;\\ \;sp_{i} \end{array}\right\}\begin{array}{c} s_{n}\;\\ \;\\ s_{i} \end{array}$$

(3) $$\left\{\left\{\Delta R_{\mathrm{horizon}\;2090}^{m_{i}}=\frac{{\text{MAV}}_{\mathrm{horizon}\;2090}^{m_{i}}.100}{{\text{MAV}}_{\mathrm{baseline}}^{m_{i}}}\right\}\begin{array}{c} \;sp_{n}\;\\ \;\;\\ sp_{i}\; \end{array}\right\}\begin{array}{c} \;s_{n}\\ \;\;\\ \;s_{i} \end{array}$$

A positive value for percent change indicates an increase in species abundance and vice versa. In addition, SR to each IHA metric was calculated as the mean ∆‐Response of each species across all sampling sites; this was calculated separately for each of the two catchments and for each of the two future time periods. Species with the most negative SR values would be most susceptible to climate change‐induced flow alteration of the respective IHA metric in that catchment.

All responses calculated above are related to a single species, while all following analyses measure responses at the species assemblage level. Each sampling site is representative of a 100‐m river reach. For each sampling site (*s*_*i*_), the species assemblage response in that river reach, ${\text{SAR}}_{r_{i}}$, to each IHA metric (*m*_*i*_) was assessed by calculating the means of the response values for all species occurring in that reach (*sp*_*1*_ to *sp*_*n*_):

(4) $${\left\{{\text{SAR}}_{r_{i}}^{m_{i}}=\text{mean}\left(\Delta R_{sp_{1}}^{m_{i}},\;\Delta R_{sp_{2}}^{m_{i}},\cdots ,\;\Delta R_{sp_{n}}^{m_{i}}\;\right)\right\}}_{s_{i}}^{s_{n}}$$

This value was separately calculated for both future time periods (i.e., horizon 2050 and horizon 2090) and each IHA metric, resulting in 10 overall values per river reach.

Although the metrics used in both catchments (Kinzig and Treene) were different, which made a direct comparison difficult, the IHA metrics inherently cocorrelated with many other metrics from the same category (Olden & Poff, 2003). Therefore, the results for both species (SRs) and species assemblage responses (SARs) are considered insensitive to the choice of the particular metrics within the same IHA category.

IHA metrics describe different aspects of key flow conditions (i.e., duration, frequency, magnitude, rate and timing) that might be unequally important for the assemblages of stream macroinvertebrates (Kuemmerlen et al., 2015; Tonkin, Stoll, Sundermann, & Haase, 2014). Therefore, the overall response of macroinvertebrate assemblages (OSARs) to flow alterations was assessed according to the mean of SAR values for all five IHA metrics in each river reach (*r*_*i*_):

(5) $${\left\{{\text{OSAR}}_{r_{i}}^{m_{\mathrm{all}}}=\text{mean}\left({\text{SAR}}_{m_{1}},\;{\text{SAR}}_{m_{2}},\;{\text{SAR}}_{m_{3}},\;{\text{SAR}}_{m_{4}},\;{\text{SAR}}_{m_{5}}\right)\right\}}_{s_{i}}^{s_{n}}$$

Therefore, all IHA metrics (*m*_all_) contributed to the overall species assemblage responses (OSARs) in each river reach. The outcome of such overall assessment (OSARs) based on partial assessments (SARs) extremely depend on the choice of the aggregation method (Langhans, Reichert, & Schuwirth, 2014).

The sensitivity of outcomes using another widely used aggregation method (the minimum aggregation method, also known as worst scenario) is shown in the supplementary material (potential worst OSARs, WOSAR). The minimum aggregation method assumes that decreased abundances caused by changes in one of the flow metrics might not be compensated by increased abundances caused by any other metrics.

All statistical analyses were carried out in R 3.3.2 (R Development Core Team, 2016). We used one‐way analysis of variance (ANOVA) for all significance tests of flow alteration and paired *t* tests to compare the means of SRs, SARs, and OSARs to flow alterations.

## 3 RESULTS

### 3.1 Potential changes in flow conditions

**Figure 3:**
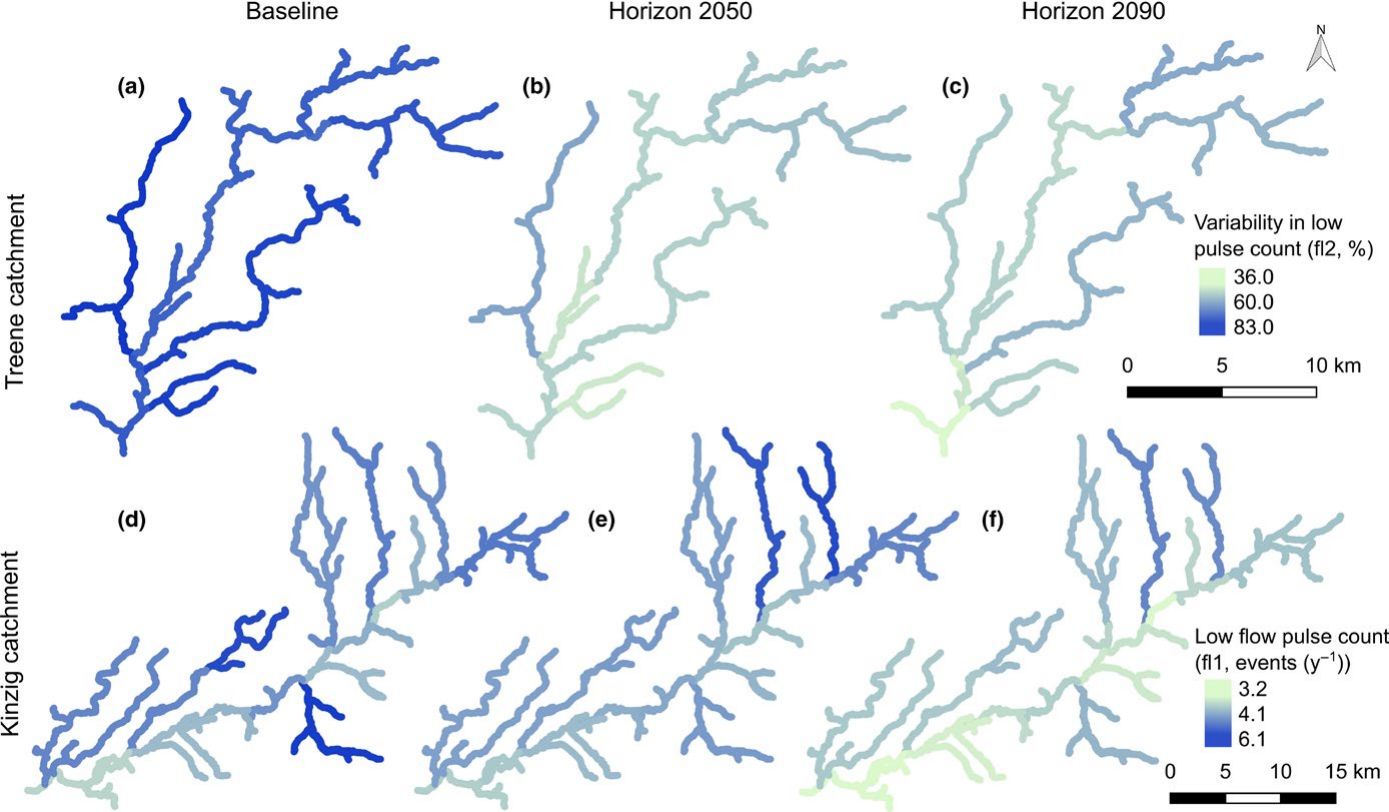
Potential changes in variability in low pulse count (fl2) in the Treene (a, b, and c) and low pulse count (fl1) in the Kinzig (d, e, and f) catchment, comparing the baseline (a and d; 1998–2017) to horizon 2050 (b and e; 2046–2065) and horizon 2090 (c and f; 2080–2099). Other changes in flow metrics in the respective catchments are shown in Figs. SF2 and SF3

**Figure 4:**
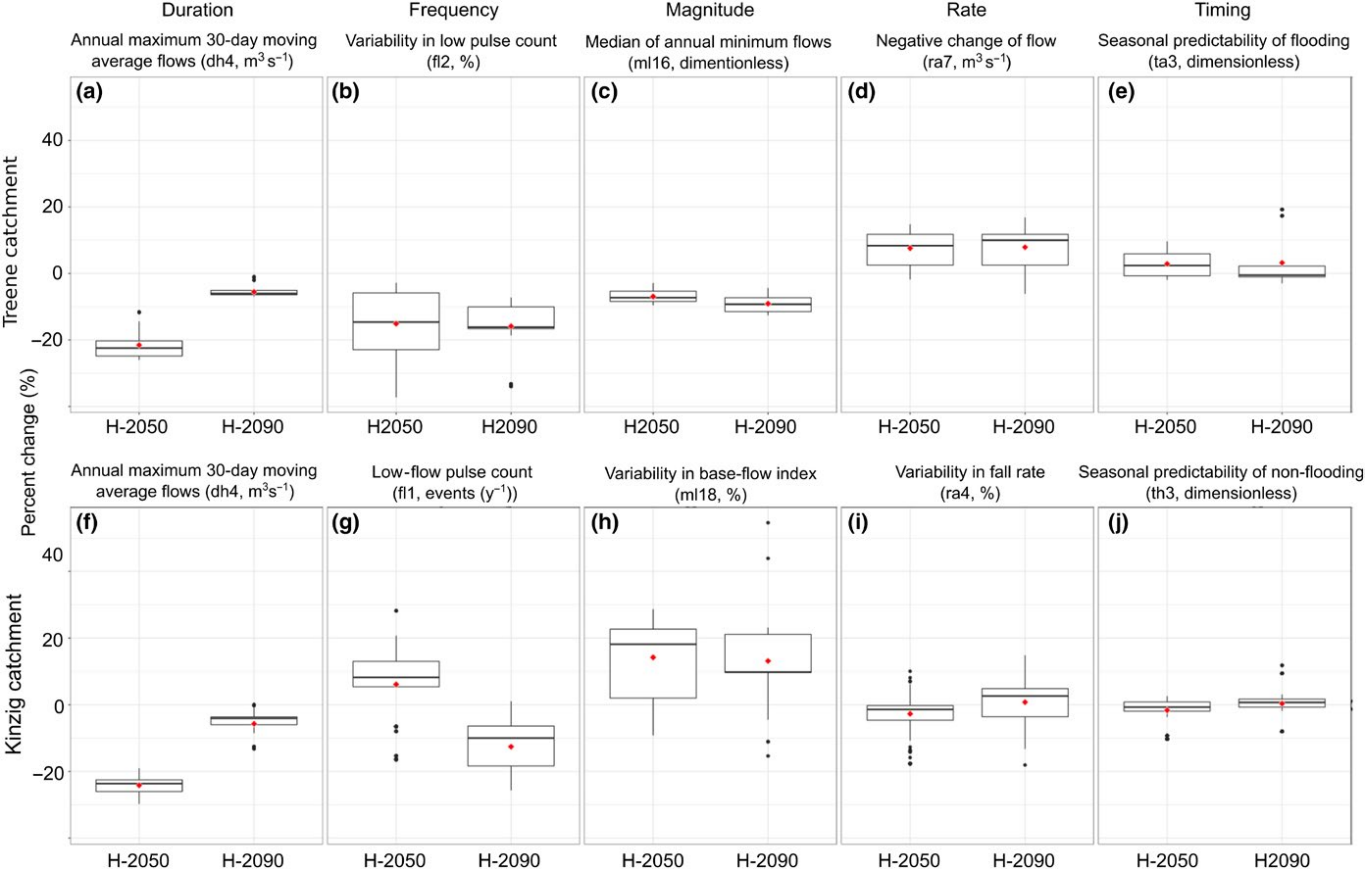
Boxplots (bar—median; red triangular—mean; box—1st and 3rd interquartile ranges) showing potential percent changes in the IHA metrics at the sampling sites of the Treene (a–e) and Kinzig (f–j) catchments for the two defined 20‐year periods of horizon 2050 (2046–2065) and horizon 2090 (2080–2099) compared to the baseline (1998–2017). For more details, see Fig. SF1

In the Kinzig catchment, climate change was predicted to most strongly affect the low‐flow conditions (Figures 3, 4, Figures S1–S3).

The variability in base‐flow index (ml18) was predicted to increase within horizon 2050 (Figure 4h, Figure S1h), while the frequency of low‐flow events was predicted to decrease in horizon 2090 (fl1, low‐flow pulse count, Figure 4g, Figure S1g). In addition, the modeled future discharge values showed a lower seasonal predictability of low‐flow events (th3, Figure 4j, Figure S1j). These predicted changes were significant for the first period, horizon 2050, similar to the two metrics describing the magnitude of high flows (dh4, annual maximum 30‐day moving average, Figure 4f, Figure S1f) and the variability of the falling rate of high‐flow events (ra4, variability of fall rate, Figure 4i, Figure S1i).

In the Treene catchment, climate change was also predicted to most strongly affect the low‐flow conditions at the sampling sites, but modeled effects were larger compared to those in the Kinzig catchment (Figure 4, Figures S1–S3). However, the modeled changes in IHA metrics describing the high‐flow conditions were less obvious but still significant (Figure 4, Figure S1). The magnitude and interannual variability of low‐flow events were predicted to markedly decrease (Figure 4b,c, Figure S1b,c) with (1) a decrease in the median annual minimum flow (ml16, lower ratios of minimum annual flows to median annual flows) and (2) a decrease in the variability of low pulse counts (fl2, lower coefficient of variation for the number of low‐flow events per year). In respect to the high‐flow events, the modeled future discharge values showed an increase in the seasonal predictability of flooding (ta3, Figure 4e, Figure S1e) and lower maximum flows, at least in the first period, horizon 2050 (dh4, maximum 30‐day maximum moving average, Figure 4a, Figure S1a, ANOVA, *p* < .05, Tukey HSD, *p* < .05).

### 3.2 Species responses (SRs)

**Figure 5:**
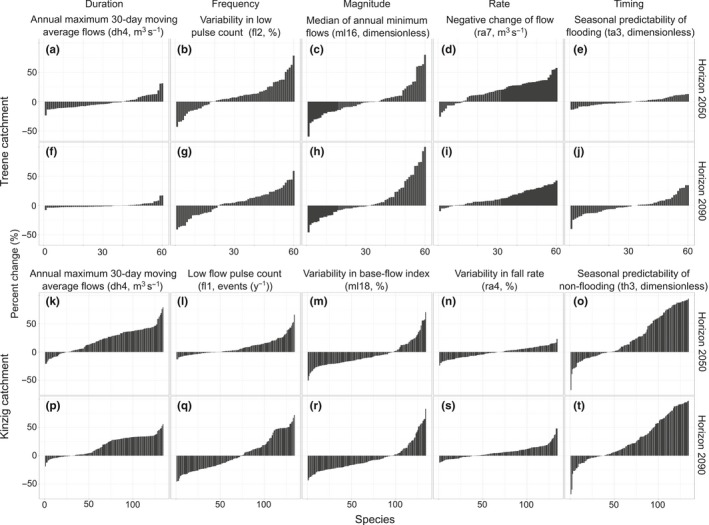
The mean response of individual species response (SRs) to each IHA metric in the Treene (60 species, a–j) and Kinzig (134 species, k–t) catchments for horizon 2050 (upper row in each catchment, a–e and k–o) and horizon 2090 (lower row in each catchment, f–j and p–t). The bars are sorted by decreasing to increasing SR

Overall, the predicted changes in SRs were larger in the Kinzig compared to the Treene catchment (Figure 5). The mean percentage change in the absolute values for all species and all metrics was significantly higher in the Kinzig compared to the Treene for both time periods. The mean change was 21.6% in the Kinzig, compared to only 13.9% in the Treene catchment for horizon 2050 (*t* test, *p* < .01), while in horizon 2090, it was 19.3% and 14.7% in the Kinzig and Treene, respectively (*t* test, *p* < .01).

In the Kinzig catchment, in accordance with the large predicted effect on the low‐flow conditions, these IHA metrics (frequency and magnitude) resulted in a decrease in abundance for a large number of species. The share of these species was significantly larger for these two IHA metrics (Figure 5q,m,r) compared to the other metrics (chi‐squared test, *p* < .05). Projected changes in the magnitude of low‐flow events (ml18) caused decreasing trends, with a percentage change of up to −50% for most of the studied species in both horizons (Figure 5m,r, 72% and 70% of species in horizon 2050 and 2090, respectively). The frequency of low‐flow events (fl1) caused greater decreases in abundances in horizon 2090, with 55% of species showing a decrease in abundance up to −46% (Figure 5q).

However, a large number of species (81% and 78% of species in horizons 2050 and 2090, respectively) were predicted to increase up to 79% in abundance and benefit from only a slight decrease in the high‐flow conditions (dh4, Figure 5k,p, mean values of each period: 7.8 for baseline, 5.9 for horizon 2050, and 7.4 for horizon 2090) and changes in flood‐free periods (th3, Figure 5o,t, 66% of species in horizon 2050 and 73% in 2090 show increased values of up to 97%, mean values of each period: 0.826 for baseline, 0.813 for horizon 2050, and 0.828 for horizon 2090). The projected changes for both IHA metrics were significant only in horizon 2050 (Figure 4f,j, Figure S1f,j, ANOVA, *p* < .05, Tukey HSD, *p* < .05).

In the Treene, the share of species with decreasing responses was also high for the metrics that were predicted to change significantly (fl2 and ml16, Figure 4b,c, Figure S1b,c and Figure 5b,c,g,h). The magnitude of SR was also highest for these metrics compared to the rest of the metrics (*t* test, *p* < .05). Furthermore, large decreasing trends were detected in response to the timing of high‐flow events (ta3).

Despite insignificant changes in the rate of change in flow events (ra7) in both horizons (Figure 4d, Figure S1d), more species (80% and 87% of species in horizons 2050 and 2090, respectively) were predicted to increase in abundance (up to 57%, Figure 5d,i) compared to all other metrics.

### 3.3 Species assemblage responses (SARs)

#### 3.3.1 Species assemblage responses (SARs) per IHA metric

**Figure 6:**
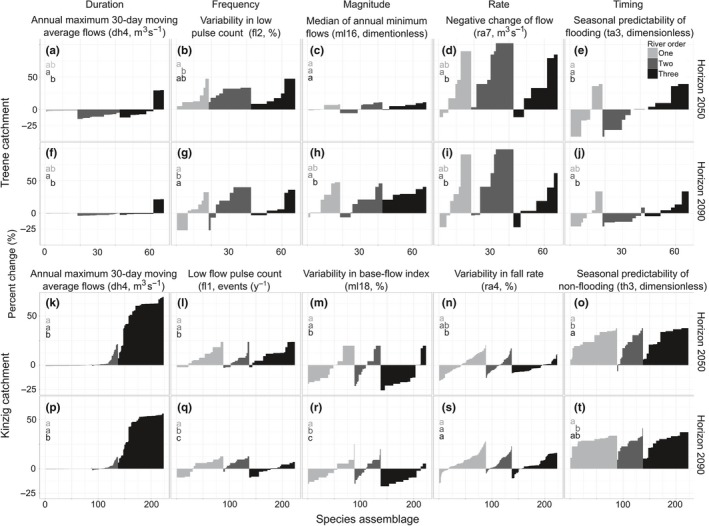
The mean response of species assemblages (SARs) at each site for each IHA metric and river order in the Treene (67 sites, a–j) and Kinzig (223 sites, k–t) catchments for horizon 2050 (upper row in each catchment, a–e and k–o) and horizon 2090 (lower row in each catchment, f–j and p–t). The characters (a, b, c, and ab) show whether the values of species assemblage responses in a river order would be significantly (*p* < .05; dissimilar characters) different from other river orders or not (similar characters)

Similar to the SRs, the predicted SARs to single IHA metrics were larger in the Kinzig compared to the Treene catchment (Figure 6, for details see Tables S2 and S3).

The mean percentage change in the absolute values over all sites (60 sites in the Treene and 223 sites in the Kinzig), and all metrics were significantly higher in the Kinzig compared to the Treene for both horizons. The mean change in absolute values was 13.8% in the Kinzig compared to only 8.0% in the Treene catchment for the horizon 2050 (*t* test, *p* < .01), and differences were smaller for the horizon 2090, with 15.6% in the Kinzig and 8.7% in the Treene catchment (*t* test, *p* < .01).

In the Kinzig, the SARs per metric shows—similar to the SR—large increases in species assemblage abundances caused by decreasing duration of high‐flow conditions (dh4), especially for the higher‐order reaches (river order three, Figure 6k,p, Figure S5a,b). The SARs to this metric were significantly higher in downstream reaches (i.e., river order three) with mostly increased AV compared to decreased values in the upstream reaches (ANOVA, *p* < .01, Tukey HSD, *p* < .01). Most increasing trends in SARs were caused by the small increased values predicted in flood‐free periods (th3, mean values of each period: 0.83 for baseline, 0.86 for horizons 2050 and 2090), while decreasing trends (Figure 6q,m,r,n, Figure S5d,e,f) were mainly caused by increased or decreased values in the low‐flow conditions (mainly increased ml18 with the following mean values of each period: 56.0% for baseline, 63.4% for horizon 2050, and 63.0% for horizon 2090, and decreased fl1 with the following mean values: 4.4 low‐flow events for baseline and 3.8 for horizon 2090, and decreased ra4 with the following mean values: 202.3 for baseline, 197.2 for horizon 2050).

The SARs of the Treene river reaches showed decreased AV to both low and high‐flow conditions described by timing, duration, and frequency of flow events (Figure 6a,e,f,g, Figure S4a,b,d,i,j). Two metrics of duration and frequency show decreased values in the future (dh4, mean values of each period: 2.6 m^3^/s for baseline, 2.0 m^3^/s for horizons 2050 and 2.4 m^3^/s for 2090, and mean values of fl2 in each period: 56.8% for baseline, 48.0% for horizons 2050, and 47.6% for 2090), while timing was projected to increase slightly (th3, mean values of each period: 0.83 for baseline, 0.86 for horizons 2050 and 2090). Decreased frequency low‐flow events (fl2, Figure 6b,g, Figure S6c,d) and rate of flow events (ra7, Figure 6d,i, Figure S6g,h, mean values of each period: 56.8% for baseline, 48.0% for horizons 2050 and 47.6% for 2090) caused most increased SARs in the Treene catchment.

Similar to the Kinzig catchment, SARs revealed increased abundances by slight (but significant) decreased values in duration of high‐flow events (dh4) in higher river orders (Figure 6a,b, Figure S4a,b), while only decreased SARs were detected in lower river orders.

#### 3.3.2 Overall species assemblage responses (OSARs, overall scenario)

Similar to the SRs and SARs, the absolute OSARs were significantly larger in the Kinzig (mean percentage change of the absolute values: 10.1% in horizon 2050 and 9.8% in horizon 2090) compared to the Treene catchment (mean percentage change of the absolute values: 5.6% in both horizons, *t* test, *p* < .01).

**Figure 7:**
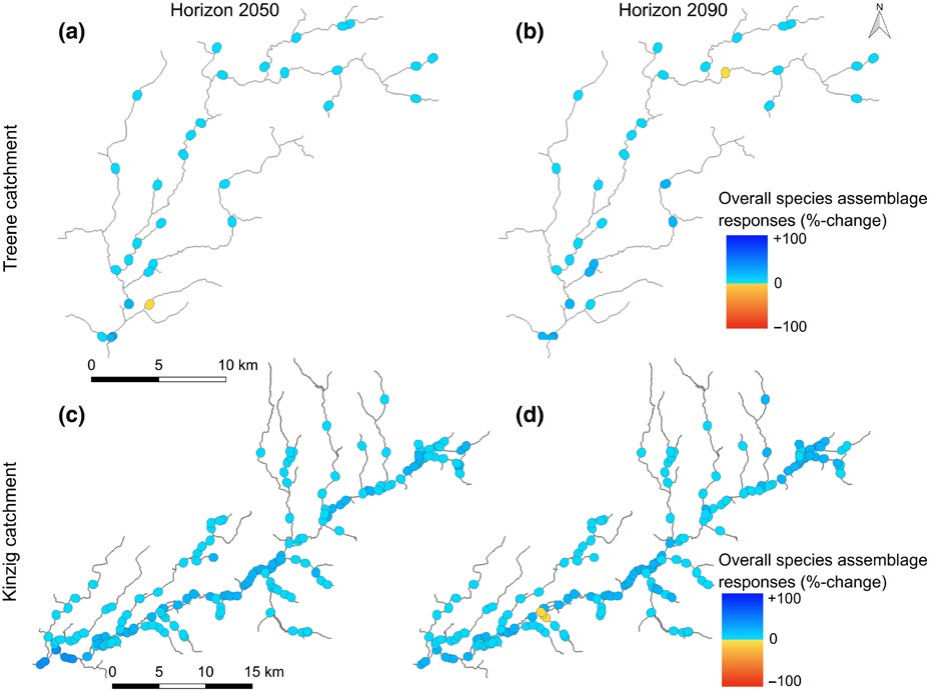
Potential overall response of species assemblages (OSARs, equation (5)) in the Treene (a and b) and Kinzig (c and d) river reaches in horizons 2050 (a and c) and 2090 (b and d), according to mean value, that is, contribution of all five IHA metrics

In the Kinzig, OSARs were predicted to be positive in all river reaches in horizon 2050, while three river reaches showed negative values in horizon 2090 (Figure 7c,d). In the Treene, positive OSARs were predicted for all river reaches except one reach in each horizon (Figure 7a,b).

## 4 DISCUSSION

Assessing the quantitative impact of possible flow alterations on SRs yielded several key findings: (1) Climate change was predicted to strongly decrease the low flows in both studied catchments; (2) the predicted increases and decreases in species abundances were not proportional to changes in flow metrics; and (3) predictions showed that species would experience decreased and increased abundances with regard to flow alterations detected by five IHA metrics in both the lowland and lower‐mountainous region. The species assemblage responses were predicted to increase at most sampling sites for most IHA metrics, which resulted in increasing OSARs in all Kinzig and 98% of Treene river reaches. These changes were significantly larger in the lower‐mountainous Kinzig compared to the lowland Treene catchment. The increased overall abundances are reasonable and can be described by the high proportion of generalist species, for example, only 26 and five habitat specialists in the Kinzig and Treene, respectively (according to Schmidt‐Kloiber & Hering, 2015). Generally, increased abundances are not identical to a better ecological status (according to regular monitoring required by the European Water Framework Directive) as the river‐type specific species might decrease in abundance while generalists or invasive species might increase strongly.

### 4.1 Flow alterations and species/assemblage abundances

We detected strong effects of climate change on low‐flow conditions in both catchments which were previously reported in in‐situ studies of European rivers (Laizé et al., 2014; Schinegger, Trautwein, Melcher, & Schmutz, 2012). For example, the lower frequency and magnitude of flow events were also detected in previous studies on the Treene catchment (Guse et al., 2015). These patterns (e.g., decreasing magnitude of low‐flow conditions) were also reported in other regions in Europe, e.g., southwestern Balkans (Papadaki et al., 2016).

**Figure 8:**
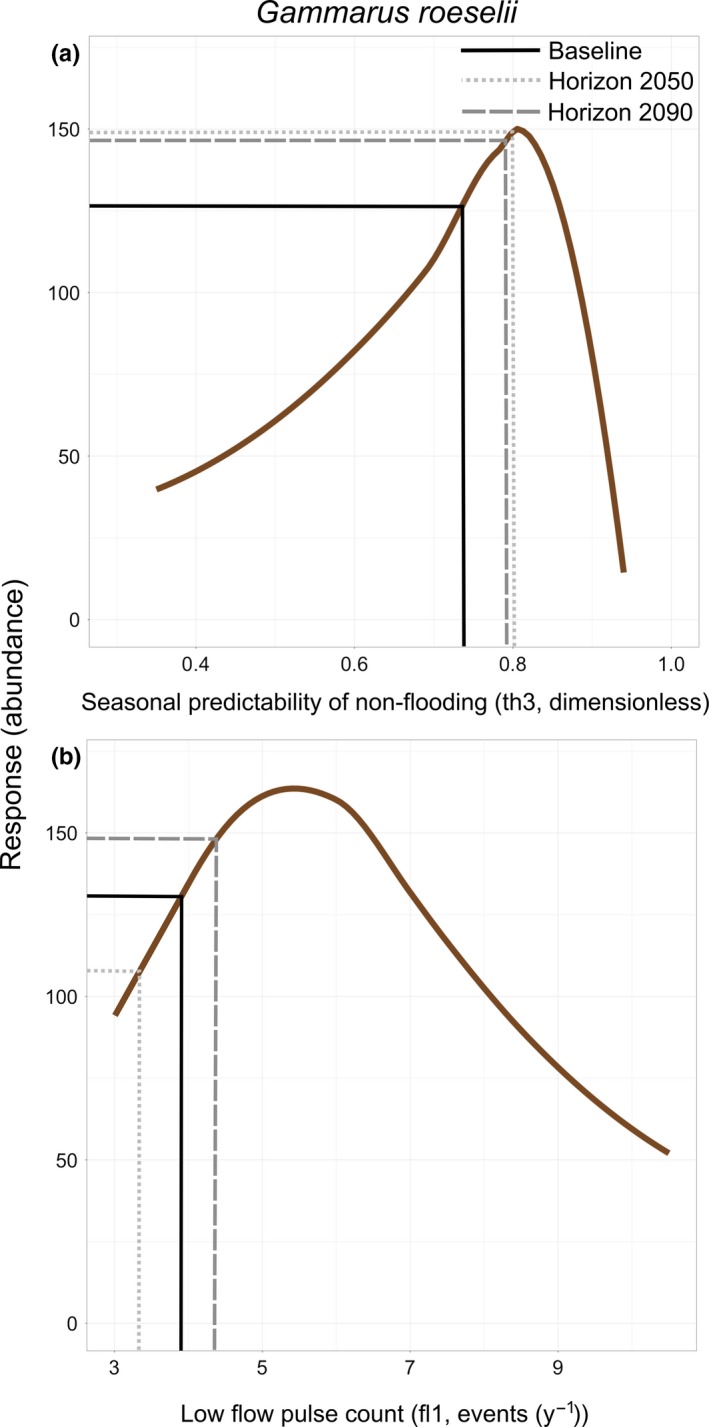
The response of *Gammarus roeselii* (Crustacea) to projected flow alterations in low‐flow pulse count (fl1, a), and seasonal predictability of nonflooding (th3, b). The (dashed) lines show the species responses to altered flow values at a random sampling site during the projected periods, compared to the baseline (solid line)

The largest and most significant changes in flow conditions were only partly reflected by species or species assemblage responses (SRs and SARs). For example, strong decreasing trends were predicted for metrics describing low‐flow conditions (frequency and magnitude of low‐flow events); however, species and assemblages showed strong responses (increased abundances) to other metrics that are projected to change less severely (e.g., Treene: rate of change in flow conditions [ra7], and Kinzig: duration of high‐flow events [dh4] and timing of low‐flow events [th3]). This revealed that even small changes in flow conditions possibly lead to strong SRs. Alternatively, slight changes in these flow conditions may result in a more suitable flow condition and subsequently a more suitable habitat that is closer to the species’ optimal preferences (e.g., *Gamarus roeselii*, Figure 8a).

It is widely reported that increasing the number of low‐flow events and discharge (e.g., downstream of dams) has negative effects on stream macroinvertebrates due to higher temperatures (Bredenhand & Samways, 2009; Dewson et al., 2007; Maheu, St‐Hilaire, Caissie, & El‐Jabi, 2016). The species assemblage responses to a decrease in the number of low flows (fl1) resulted in an increase in species assemblage abundances (Figure 6) which is expected ecologically.

Moreover, the predicted decrease in abundances caused by fewer low‐flow events (fl1) in horizon 2090 might be due to the sensitive range of flow conditions, that is, minimum values, which will be affected most by climate change. An example of the modeled predictive relationship of *G. roeselii* (Trichoptera, Figure 8b) shows how species preferences to specific ranges of low‐flow frequencies (fl1) might cause their abundances to decrease. The peak preference values of about five annual low‐flow events indicate that the disturbances caused by more frequent low‐flow events lead to negative responses in this species. So this species might prefer low‐flow conditions in certain stages of their life cycle, for example, for hatching, laying eggs, or emergence (Lancaster & Downes, 2010). However, the positive responses of SARs to fewer low‐flow events (fl1, Figure 6q) show that they favor the projected decrease in low‐flow conditions.

Furthermore, the decreased variability and frequency of low‐flow events observed in our climate models for both central European catchments, that is, less stress on the species in that respect, resulted in increasing abundances of both species and assemblages of stream macroinvertebrates. However, increasing frequencies of low‐flow events, and hence, decreasing species diversity were reported in other regions (Brooks & Haeusler, 2016; Chessman, 2013, 2015; Dewson et al., 2007; Leigh & Datry, 2016). This reveals the importance of spatial scale of climate‐change studies and regional differences in the type of responses.

Some studies reported changes up to −100% in species richness due to the loss of climatically suitable habitats caused by warming climates (Domisch et al., 2013) or extinctions (according to species probability of occurrences) by changes in flow and/or temperature (Pyne & Poff, 2017). Our findings show that the SRs barely exceeded percent‐change values ranging smaller than −50% and larger than +50% in the Treene and Kinzig catchments. We were only looking on the effect of climate change on stream macroinvertebrates via its effect on flow conditions. Even when generalists potentially will benefit from the flow alterations, other environmental variables that are changing with climate change may counteract. This reveals that flow alterations, as a single stressor, might not lead to catchment‐scale extinctions among the studied species, and hence, extinctions or more severe decreasing trends in species diversity may depend on additional effects from other environmental stressors (e.g., temperature) or decreasing habitat suitability (Dewson et al., 2007; Pyne & Poff, 2017). Furthermore, differences in the taxonomic resolution, variables, and time scales or the smaller spatial scale with much finer resolution in our study, compared to other studies, might be the reason for the lower predicted impacts of climate change on stream macroinvertebrates observed in this study. Furthermore, our limited understanding of biotic interactions hinders attempts to add these factors to observed relationships.

### 4.2 Effects of flow alterations on each catchment

We observed stronger potential flow alterations in the Kinzig compared to the Treene catchment, probably due to different catchment characteristics. The Treene is a lowland groundwater‐dominated river with low hydrological gradients (Guse et al., 2015; Kiesel, Fohrer, Schmalz, & White, 2010; Pfannerstill, Guse, & Fohrer, 2014) which showed low ranges of flow alterations; however, the Kinzig is a precipitation‐driven lower‐mountainous river with high hydrological gradients which will be highly affected by the climate change‐induced flow alterations.

The observed higher magnitude of SRs, SARs, and OSARs in the Kinzig compared to the Treene catchment might be linked to (1) the differences in flow regimes and catchment characteristics between the lowland (Treene) and lower‐mountainous region (Kinzig, Table 1), and (2) different effects of climate change on flow regime in each region (lowland vs. lower‐mountainous region) according to climate models (Figure 4). Yet another possible explanation is the lower hydraulic and hydrological gradient in the Treene compared to the Kinzig, which lead to higher impact of even small flow alterations on stream macroinvertebrates responses. This confirmed the results of several studies (Buisson & Grenouillet, 2009; Fenoglio, Bo, Cucco, Mercalli, & Malacarne, 2010; Poff, Pyne, Bledsoe, Cuhaciyan, & Carlisle, 2010), which reported that both species and assemblages of freshwater biota are likely to respond stronger in regions with higher streamflows (discharge) and stronger hydraulic and hydrological gradient, alternatively, because flow alterations are stronger in rivers with strong hydrological gradient and high streamflows (e.g., in the steeper lower‐mountainous Kinzig). Possibly, strong flow alterations—representing hydrological disturbances, create environmental filters for species occurrences, mainly through changing geomorphic and physical habitat conditions (Rolls et al., 2017). Moreover, the higher channel slope in the Kinzig and hence the higher flow velocity, especially in the first‐order headwaters, has a stronger effect on the shear stress. If the shear stress at high flows decreases in the Kinzig (e.g., from 20 to 10 Nm^2^), this might have a tremendous effect on generalist species that cannot stand high shear stress while a small decrease in shear stress (e.g., from 5 to 2.5 Nm^2^) in the lowland Treene is just reducing an already nondisturbing stress to an even lower stress.

Furthermore, the lowland Treene, a groundwater‐dominated river with low variability in flow conditions, may respond slower to climate change compared to the Kinzig. The stable flow regime may cause more generalists and fewer specialists to occur in the Treene (only five habitat specialists) compared to a higher proportion of specialists in the Kinzig (26 habitat specialists, according to Schmidt‐Kloiber & Hering, 2015). Therefore, the species assemblages of the Treene reaches might cope better with the flow alterations compared to the Kinzig catchment.

#### 4.2.1 Effects of flow alterations on rivers of different size

In this catchment‐scale study, the response of stream macroinvertebrates to flow alterations varied with river order, and most positive responses were detected in higher river orders, while most decreased abundances were detected in the lower river orders and upstream area. Headwater systems are critical areas for stream macroinvertebrates habitats (Meyer & Wallace, 2001) because they are subject to more temporal and spatial variation (Gomi, Sidle, & Richardson, 2002). Hence, projected changes in the upstream area with lower discharge magnitudes will affect the species more than changes in the downstream area. For example, a slight but significant decrease in duration of high‐flow events (dh4) in both catchments was predicted to affect the communities in upstream reaches more than in downstream reaches (Figure 6a,f,k,p, Figures S4, S5), as the increased abundances were detected in only downstream reaches (river order three) in both horizons. This means that the communities that inhabit the higher‐order reaches would benefit from climate change, and the predicted flow conditions would be closer to species’ flow preferences.

The increase in SARs (Figure 6k) caused by the decrease in peak flows in the Kinzig (Figure 4f horizon 2050) might be due to the fact that many species also occurring in the lowlands (i.e., generalists) suffer from high flows and will increase in abundance if the peak flows decrease, while the few specialists adapted to these high flows decrease in abundance (species with negative values in Figure 5k). This is supported by the fact that the increase in abundance due to the reduced peak flows is much lower in the first‐order reaches compared to the larger third‐order reaches. Alternatively, because slope is very high in the first‐order reaches, only the rheophilic specialists occur in headwaters. The specialists will not benefit but suffer from a decrease in high flows, while the generalists occur usually in the third‐order reaches where the observed decrease in peak flows favors them.

Although decreasing high‐flow events in the higher‐order reaches will decrease species downstream drift (Death, 2008), it might affect species through higher temperatures (Pyne & Poff, 2017) and lower oxygen content (Allan & Castillo, 2007). This ecological effect can also be well described by species increased abundances in response to significant decrease in low‐flow conditions (fl1 in Kinzig, Figures 4g and 6l,q). These increasing trends show the vulnerability of species to, for example, prolonged low‐flow conditions, which have been most often explored in recent years (Leigh, 2013; Leigh & Datry, 2016; Walters, 2016).

### 4.3 Outlook

Overall, invertebrate abundance was predicted to increase due to climate change‐induced flow alterations (which we consider surprising). Although the species abundances can be affected by potential changes in other environmental variables (e.g., temperature), the observed increase in overall species assemblage abundances might be due to the fact that generalists will benefit from the flow alterations. However, the sensitive species of conservation interest are probably among the ones that will decrease in abundance (e.g., indicated by the much lower overall increase in abundances in the headwaters); therefore, further studies including information on the taxa groups increasing and decreasing in abundance will give more information on this.

Effects of projected flow alterations might be manifested as either changes in community structure and composition of aquatic fauna or loss of ecosystem functioning and services (Laizé et al., 2014). Our study suggests that changes in flow conditions would lead to a variety of responses in stream macroinvertebrates. These species are indicators of ecosystem health. Furthermore, healthy aquatic ecosystems provide ecosystem services such as clean drinking water (Brisbane Declaration, 2007). Analyzing the responses of individual species to flow alterations might further reveal whether SRs to flow alterations can be considered as ecologically positive or negative. For example, increased abundances of, for example, *Dugesia* sp. might be ecologically negative as it is known to be the indicator of low water quality (Johnson, Wiederholm, & Rosenberg, 1993).

Upscaling catchment‐scale spatial variation in SRs to flow alterations and the subsequent effects on community structure and composition can provide insights into potential shifts across broad climatic gradients at larger spatial scales (Campbell, Winterbourn, Cochrane, & Mcintosh, 2015).

Although the few studies that assessed the effects of multiple stressors on stream macroinvertebrates reported higher impacts of some stressors (e.g., land‐use) other than flow (Kuemmerlen et al., 2014, 2015), flow alteration is reported to be among the most important variables affecting the species of stream macroinvertebrates (Poff, Tharme, & Arthington, 2017). The method introduced in this study, that is, the quantitative assessment of flow‐ecology relationships, can be applied to any specific IHA metric according to research interests (e.g., high‐/low‐flow conditions, extreme events, zero‐flow days) or any quantitative environmental variable (e.g., temperature) to assess the effects of global changes on river ecosystems. It can also be applied and modified for use in other regions and at different spatial and temporal scales. We suggest further quantitative flow alteration—species abundance relationship studies in other regions, for example, Mediterranean region or Alpine territory, where flow conditions might change differently than in central Europe.

## CONFLICT OF INTEREST

None declared.
