# Supplementary material for: Projected effects of Climate‐change‐induced flow alterations on stream macroinvertebrate abundances
Source: Ecol Evol. 2018 Feb 22;8(6):3393–409. doi: 10.1002/ece3.3907 (PMC5869304; doi:10.1002/ece3.3907)
Supplement: Supplementary file 1 [file ECE3-8-3393-s001.docx]

# Supplementary material

## Potential changes in flow conditions

The flow conditions during the baseline period (1998 – 2017) were compared to the two projected periods of horizons 2050 (2046 – 2065) and 2090 (2080 – 2099). Figures S2 and S3 show the potential changes in flow conditions for each metric in the Treene and Kinzig catchments.

## Potential worst overall species assemblage responses (WOSARs, worst scenario)

The worst scenario assumes that the IHA metric with the worst impact on macroinvertebrate communities would be the determining factor in the species response to flow alteration, regardless of whether positive values were detected for the other metrics. Therefore, the lowest value of ${CR}_{r_{i}}$ among the five metrics represented the overall species assemblage response (WOSAR) of individual river reaches in each horizon:

| $\left\{ {WOCR}_{r_{i}}^{m_{all}}=MIN\left( {SAR}_{r_{i}}^{m_{1}}, {SAR}_{r_{i}}^{m_{2}}, {SAR}_{r_{i}}^{m_{3}}, {SAR}_{r_{i}}^{m_{4}}, {SAR}_{r_{i}}^{m_{5}} \right) \right\}_{s_{i}}^{s_{n}}$ | Eq. 6 |
| --- | --- |

In addition, we tested how proportional weights of each IHA metric, given their potential impact, would affect the outcome. Furthermore, we analysed the results separately for three river orders in each river catchment to assess whether different species responses to flow alteration would be expected in different river orders.

This method obviously resulted in a different pattern for macroinvertebrate species assemblage responses in river reaches in both catchments (Fig. S6). The differences between the two methods in both horizons and both catchments were assessed to be significant (ANOVA, p < 0.01, Tukey HSD, p < 0.01).

Similar to the SARs, the absolute WOSARs were significantly larger in the Kinzig (13.4% in both horizons) compared to the Treene catchment (9.9% in horizon 2050 and 9.3% in horizon 2090, t-test, p<0.01).

In the Kinzig, positive WOSARs were only predicted for 3% of the river reaches in horizon 2050 (Fig. S6g) and 14% in horizon 2090 (Fig. S6h). Negative WOSARs meant that at least one out of the five metrics predicted a decrease in mean species’ abundances and were most often due to changes in low flow condition metrics (ml18). The negative WOSARs in 56% of the river reaches in horizon 2050 and 65% of the river reaches in horizon 2090 were related to changes in the magnitude of low-flow events (ml18, Fig. S6e, S6f). The magnitude of flow events is widely reported to have strong effects on macroinvertebrate assemblage metrics (Monk *et al.*, 2006; Poff & Zimmerman, 2010), e.g., through lower base flow, higher temperature and lower oxygen content.

In the Treene, negative WOSARs were predicted for all river reaches for both horizons (Fig. S6c, S6d). The timing of high-flow events (ta3, 45% in horizon 2050 and 58% in 2090) and duration of high flow events (dh4, 52% in horizon 2050) were the most frequent reasons for negative WOSARs for one or both horizons (Fig. S6a, S6b).

In the Kinzig catchment, the magnitude of low flow events (ml18) caused decreased abundances mainly in higher river orders amd main stream in both horizons, while frequency of low flow events (fl1) and duration of high flow events (dh4) caused decreased abundances in far upstream region in horizon 2050 and 2090, respectively.

In the Treene catchment, decreased abundances of the upstream area were mostly caused by timing of high flow events (ta3) and duration of high flow events (dh4) in horizon 2050, or timing of high flow events (ta3) and frequency of low flow events (fl2) in horizon 2090.

## References

Monk WA, Wood PJ, Hannah DM, Wilson DA, Extence CA, Chadd RP (2006) Flow variability and macroinvertebrate community response within riverine systems. River Research and Applications*,* **22**, 595-615.

Poff NL, Zimmerman JKH (2010) Ecological responses to altered flow regimes: a literature review to inform the science and management of environmental flows. Freshwater Biology*,* **55**, 194–205.

## Tables

**Table S1.** List of all 60 and 134 species of stream macroinvertebrates in respectively the Treene and Kinzig catchments, respectively, and the author and higher taxonomical unit.

| **Species** | **Author** | **Higher taxa** | **Treene** | **Kinzig** | **Within sample AUC** | | | | | | | | |  | **Cross-validated AUC** | | | | | | | | |  |
| --- | --- | --- | --- | --- | --- | --- | --- | --- | --- | --- | --- | --- | --- | --- | --- | --- | --- | --- | --- | --- | --- | --- | --- | --- |
|  |  |  |  |  | **Duration** | | **Frequency** | | **Magnitude** | | **Rate** | | **Timing** | | **Duration** | | **Frequency** | | **Magnitude** | | **Rate** | | **Timing** | |
|  |  |  |  |  | **Treene** | **Kinzig** | **Treene** | **Kinzig** | **Treene** | **Kinzig** | **Treene** | **Kinzig** | **Treene** | **Kinzig** | **Treene** | **Kinzig** | **Treene** | **Kinzig** | **Treene** | **Kinzig** | **Treene** | **Kinzig** | **Treene** | **Kinzig** |
| *Pisidium amnicum* | O.F. MÜLLER, 1774 | Bivalvia | X | X | 0.94 | 0.91 | 0.89 | 0.86 | 0.93 | 0.89 | 0.88 | 0.90 | 0.80 | 0.84 | 0.95 | 0.91 | 0.91 | 0.86 | 0.91 | 0.87 | 0.88 | 0.88 | 0.78 | 0.84 |
| *Pisidium casertanum* | POLI, 1791 | Bivalvia | - | X | - | 0.85 | - | 0.86 | - | 0.88 | - | 0.92 | - | 0.87 | - | 0.89 | - | 0.83 | - | 0.90 | - | 0.91 | - | 0.86 |
| *Pisidium subtruncatum* | MALM, 1855 | Bivalvia | X | X | 0.95 | 0.82 | 0.91 | 0.79 | 0.94 | 0.85 | 0.88 | 0.87 | 0.78 | 0.82 | 0.96 | 0.86 | 0.92 | 0.84 | 0.94 | 0.90 | 0.92 | 0.90 | 0.76 | 0.85 |
| *Pisidium supinum* | A. SCHMIDT, 1851 | Bivalvia | X | - | 0.95 | - | 0.89 | - | 0.95 | - | 0.89 | - | 0.82 | - | 0.94 | - | 0.90 | - | 0.94 | - | 0.90 | - | 0.80 | - |
| *Sphaerium corneum* | (LINNAEUS, 1758 | Bivalvia | X | X | 0.98 | 0.92 | 0.91 | 0.88 | 0.96 | 0.93 | 0.89 | 0.92 | 0.75 | 0.87 | 0.96 | 0.95 | 0.93 | 0.91 | 0.94 | 0.95 | 0.88 | 0.94 | 0.72 | 0.89 |
| *Elmis aenea* | MÜLLER, 1806 | Coleoptera | X | X | 0.97 | 0.88 | 0.91 | 0.84 | 0.92 | 0.88 | 0.88 | 0.90 | 0.80 | 0.84 | 0.95 | 0.91 | 0.90 | 0.87 | 0.93 | 0.93 | 0.87 | 0.93 | 0.76 | 0.88 |
| *Elmis maugetii* | LATREILLE, 1798 | Coleoptera | X | X | 0.97 | 0.88 | 0.91 | 0.84 | 0.91 | 0.89 | 0.87 | 0.88 | 0.82 | 0.84 | 0.96 | 0.92 | 0.91 | 0.88 | 0.93 | 0.93 | 0.86 | 0.93 | 0.78 | 0.88 |
| *Elmis rietscheli* | STEFFAN, 1958 | Coleoptera | X | X | 0.97 | 0.94 | 0.88 | 0.88 | 0.90 | 0.94 | 0.88 | 0.94 | 0.82 | 0.89 | 0.94 | 0.89 | 0.90 | 0.85 | 0.92 | 0.91 | 0.90 | 0.94 | 0.75 | 0.87 |
| *Elmis rioloides* | KUWERT, 1890 | Coleoptera | X | X | 0.97 | 0.94 | 0.89 | 0.88 | 0.89 | 0.94 | 0.88 | 0.94 | 0.84 | 0.89 | 0.96 | 0.88 | 0.90 | 0.85 | 0.92 | 0.90 | 0.87 | 0.93 | 0.78 | 0.89 |
| *Elodes minuta* | LINNAEUS, 1767 | Coleoptera | X | - | 0.95 | - | 0.91 | - | 0.97 | - | 0.86 | - | 0.69 | - | 0.95 | - | 0.92 | - | 0.97 | - | 0.83 | - | 0.66 | - |
| *Hydraena dentipes* | GERMAR, 1844 | Coleoptera | - | X | - | 0.88 | - | 0.81 | - | 0.89 | - | 0.92 | - | 0.76 | - | 0.83 | - | 0.79 | - | 0.90 | - | 0.86 | - | 0.79 |
| *Hydraena gracilis* | GERMAR, 1824 | Coleoptera | - | X | - | 0.85 | - | 0.86 | - | 0.87 | - | 0.89 | - | 0.86 | - | 0.90 | - | 0.87 | - | 0.89 | - | 0.92 | - | 0.88 |
| *Hydraena minutissima* | STEPHENS, 1829 | Coleoptera | - | X | - | 0.81 | - | 0.78 | - | 0.85 | - | 0.88 | - | 0.77 | - | 0.83 | - | 0.81 | - | 0.86 | - | 0.87 | - | 0.83 |
| *Limnius perrisi* | DUFOUR, 1843 | Coleoptera | - | X | - | 0.90 | - | 0.88 | - | 0.90 | - | 0.90 | - | 0.86 | - | 0.93 | - | 0.90 | - | 0.93 | - | 0.94 | - | 0.89 |
| *Limnius volckmari* | PANZER, 1793 | Coleoptera | X | X | 0.93 | 0.89 | 0.91 | 0.84 | 0.92 | 0.88 | 0.90 | 0.88 | 0.81 | 0.85 | 0.95 | 0.91 | 0.91 | 0.88 | 0.94 | 0.92 | 0.91 | 0.93 | 0.80 | 0.89 |
| *Orectochilus villosus* | MÜLLER, 1776 | Coleoptera | X | X | 0.93 | 0.81 | 0.91 | 0.82 | 0.93 | 0.83 | 0.81 | 0.85 | 0.72 | 0.82 | 0.95 | 0.87 | 0.90 | 0.87 | 0.92 | 0.88 | 0.80 | 0.89 | 0.71 | 0.86 |
| *Oulimnius tuberculatus* | MÜLLER, 1806 | Coleoptera | X | X | 0.95 | 0.88 | 0.91 | 0.86 | 0.94 | 0.90 | 0.83 | 0.91 | 0.78 | 0.84 | 0.94 | 0.91 | 0.92 | 0.87 | 0.93 | 0.91 | 0.82 | 0.93 | 0.71 | 0.87 |
| *Platambus maculatus* | LINNAEUS, 1758 | Coleoptera | - | X | - | 0.78 | - | 0.84 | - | 0.85 | - | 0.82 | - | 0.75 | - | 0.89 | - | 0.86 | - | 0.89 | - | 0.86 | - | 0.81 |
| *Asellus aquaticus* | LINNAEUS, 1758 | Crustacea | X | X | 0.97 | 0.89 | 0.88 | 0.85 | 0.91 | 0.88 | 0.87 | 0.88 | 0.75 | 0.86 | 0.98 | 0.93 | 0.91 | 0.89 | 0.94 | 0.92 | 0.90 | 0.92 | 0.79 | 0.90 |
| *Gammarus fossarum* | KOCH in PANZER, 1836 | Crustacea | - | X | - | 0.92 | - | 0.91 | - | 0.93 | - | 0.94 | - | 0.91 | - | 0.96 | - | 0.92 | - | 0.95 | - | 0.97 | - | 0.92 |
| *Gammarus pulex* | LINNAEUS, 1758 | Crustacea | X | X | 0.98 | 0.94 | 0.93 | 0.90 | 0.96 | 0.94 | 0.88 | 0.93 | 0.74 | 0.90 | 0.97 | 0.95 | 0.93 | 0.92 | 0.95 | 0.95 | 0.88 | 0.95 | 0.73 | 0.92 |
| *Gammarus roeselii* | GERVAIS, 1835 | Crustacea | - | X | - | 0.94 | - | 0.90 | - | 0.94 | - | 0.94 | - | 0.91 | - | 0.96 | - | 0.93 | - | 0.96 | - | 0.96 | - | 0.93 |
| *Proasellus coxalis* | DOLLFUS, 1892 | Crustacea | X | - | 0.93 | - | 0.87 | - | 0.93 | - | 0.89 | - | 0.77 | - | 0.91 | - | 0.92 | - | 0.91 | - | 0.88 | - | 0.72 | - |
| *Atherix ibis* | FABRICIUS, 1798 | Diptera | X | X | 0.97 | 0.94 | 0.92 | 0.88 | 0.92 | 0.91 | 0.83 | 0.94 | 0.73 | 0.92 | 0.94 | 0.94 | 0.92 | 0.88 | 0.95 | 0.87 | 0.91 | 0.91 | 0.83 | 0.88 |
| *Atrichops crassipes* | MEIGEN, 1820 | Diptera | - | X | - | 0.89 | - | 0.86 | - | 0.87 | - | 0.90 | - | 0.87 | - | 0.88 | - | 0.86 | - | 0.82 | - | 0.85 | - | 0.85 |
| *Chironomus riparius* | MEIGEN, 1804 | Diptera | - | X | - | 0.97 | - | 0.90 | - | 0.95 | - | 0.95 | - | 0.93 | - | 0.96 | - | 0.86 | - | 0.91 | - | 0.90 | - | 0.88 |
| *Prodiamesa olivacea* | MEIGEN, 1818 | Diptera | X | X | 0.96 | 0.86 | 0.88 | 0.85 | 0.93 | 0.85 | 0.84 | 0.88 | 0.77 | 0.88 | 0.95 | 0.91 | 0.94 | 0.88 | 0.92 | 0.90 | 0.88 | 0.92 | 0.78 | 0.90 |
| *Ptychoptera paludosa* | MEIGEN, 1804 | Diptera | X | - | 0.94 | - | 0.93 | - | 0.94 | - | 0.90 | - | 0.71 | - | 0.90 | - | 0.95 | - | 0.90 | - | 0.89 | - | 0.70 | - |
| *Simulium argyreatum* | MEIGEN, 1838 | Diptera | - | X | - | 0.94 | - | 0.82 | - | 0.90 | - | 0.94 | - | 0.85 | - | 0.92 | - | 0.81 | - | 0.86 | - | 0.91 | - | 0.83 |
| *Simulium cryophilum* | RUBZOV, 1959 | Diptera | - | X | - | 0.91 | - | 0.87 | - | 0.89 | - | 0.89 | - | 0.83 | - | 0.92 | - | 0.87 | - | 0.90 | - | 0.91 | - | 0.85 |
| *Simulium equinum* | LINNAEUS, 1758 | Diptera | X | - | 0.97 | - | 0.91 | - | 0.94 | - | 0.81 | - | 0.72 | - | 0.94 | - | 0.94 | - | 0.94 | - | 0.82 | - | 0.71 | - |
| *Simulium ornatum* | MEIGEN, 1818 | Diptera | X | X | 0.97 | 0.92 | 0.91 | 0.83 | 0.95 | 0.91 | 0.83 | 0.92 | 0.75 | 0.89 | 0.94 | 0.93 | 0.95 | 0.86 | 0.93 | 0.93 | 0.84 | 0.91 | 0.74 | 0.87 |
| *Simulium variegatum* | MEIGEN, 1818 | Diptera | - | X | - | 0.93 | - | 0.82 | - | 0.89 | - | 0.94 | - | 0.85 | - | 0.91 | - | 0.81 | - | 0.84 | - | 0.90 | - | 0.83 |
| *Simulium vernum* | MACQUART, 1826 | Diptera | - | X | - | 0.86 | - | 0.84 | - | 0.88 | - | 0.88 | - | 0.86 | - | 0.89 | - | 0.86 | - | 0.91 | - | 0.90 | - | 0.87 |
| *Baetis alpinus* | PICTET, 1843-1845 | Ephemeroptera | - | X | - | 0.88 | - | 0.84 | - | 0.91 | - | 0.90 | - | 0.87 | - | 0.89 | - | 0.87 | - | 0.87 | - | 0.90 | - | 0.87 |
| *Baetis atrebatinus* | LEACH, 1815 | Ephemeroptera | X | - | 0.95 | - | 0.91 | - | 0.86 | - | 0.83 | - | 0.78 | - | 0.96 | - | 0.92 | - | 0.93 | - | 0.86 | - | 0.73 | - |
| *Baetis fuscatus* | LINNAEUS, 1761 | Ephemeroptera | X | X | 0.95 | 0.92 | 0.92 | 0.91 | 0.86 | 0.93 | 0.83 | 0.91 | 0.77 | 0.86 | 0.96 | 0.95 | 0.93 | 0.92 | 0.92 | 0.95 | 0.85 | 0.94 | 0.73 | 0.88 |
| *Baetis lutheri* | MÜLLER-LIEBENAU, 1967 | Ephemeroptera | - | X | - | 0.89 | - | 0.87 | - | 0.89 | - | 0.90 | - | 0.86 | - | 0.93 | - | 0.87 | - | 0.90 | - | 0.92 | - | 0.88 |
| *Baetis muticus* | LINNAEUS, 1758 | Ephemeroptera | - | X | - | 0.94 | - | 0.84 | - | 0.92 | - | 0.94 | - | 0.88 | - | 0.94 | - | 0.85 | - | 0.92 | - | 0.94 | - | 0.89 |
| *Baetis niger* | LINNAEUS, 1761 | Ephemeroptera | - | X | - | 0.90 | - | 0.82 | - | 0.86 | - | 0.92 | - | 0.87 | - | 0.90 | - | 0.85 | - | 0.90 | - | 0.93 | - | 0.88 |
| *Baetis rhodani* | PICTET, 1843-1845 | Ephemeroptera | X | X | 0.95 | 0.90 | 0.90 | 0.88 | 0.91 | 0.90 | 0.84 | 0.90 | 0.72 | 0.89 | 0.95 | 0.95 | 0.93 | 0.92 | 0.93 | 0.95 | 0.89 | 0.95 | 0.70 | 0.93 |
| *Baetis vernus* | CURTIS, 1834 | Ephemeroptera | X | X | 0.95 | 0.93 | 0.91 | 0.88 | 0.86 | 0.92 | 0.83 | 0.92 | 0.78 | 0.89 | 0.96 | 0.94 | 0.92 | 0.91 | 0.91 | 0.93 | 0.86 | 0.93 | 0.75 | 0.91 |
| *Caenis horaria* | LINNAEUS, 1758 | Ephemeroptera | X | - | 0.94 | - | 0.93 | - | 0.92 | - | 0.84 | - | 0.62 | - | 0.94 | - | 0.92 | - | 0.93 | - | 0.86 | - | 0.63 | - |
| *Caenis rivulorum* | EATON, 1884 | Ephemeroptera | X | - | 0.97 | - | 0.92 | - | 0.91 | - | 0.84 | - | 0.76 | - | 0.95 | - | 0.92 | - | 0.94 | - | 0.86 | - | 0.74 | - |
| *Centroptilum luteolum* | MÜLLER, 1776 | Ephemeroptera | - | X | - | 0.91 | - | 0.87 | - | 0.94 | - | 0.89 | - | 0.89 | - | 0.93 | - | 0.88 | - | 0.94 | - | 0.92 | - | 0.91 |
| *Ecdyonurus dispar* | CURTIS, 1834 | Ephemeroptera | - | X | - | 0.92 | - | 0.87 | - | 0.91 | - | 0.93 | - | 0.87 | - | 0.90 | - | 0.86 | - | 0.88 | - | 0.91 | - | 0.85 |
| *Ecdyonurus macani* | THOMAS & SOWA, 1970 | Ephemeroptera | - | X | - | 0.87 | - | 0.81 | - | 0.86 | - | 0.84 | - | 0.85 | - | 0.85 | - | 0.82 | - | 0.86 | - | 0.83 | - | 0.85 |
| *Ecdyonurus submontanus* | LANDA, 1969 | Ephemeroptera | - | X | - | 0.90 | - | 0.85 | - | 0.90 | - | 0.92 | - | 0.90 | - | 0.90 | - | 0.84 | - | 0.90 | - | 0.92 | - | 0.87 |
| *Ecdyonurus torrentis* | KIMMINS, 1942 | Ephemeroptera | - | X | - | 0.88 | - | 0.83 | - | 0.89 | - | 0.87 | - | 0.85 | - | 0.92 | - | 0.87 | - | 0.92 | - | 0.93 | - | 0.88 |
| *Ecdyonurus venosus* | FABRICIUS, 1775 | Ephemeroptera | - | X | - | 0.91 | - | 0.88 | - | 0.92 | - | 0.94 | - | 0.86 | - | 0.90 | - | 0.84 | - | 0.90 | - | 0.92 | - | 0.88 |
| *Electrogena affinis* | EATON, 1886 | Ephemeroptera | - | X | - | 0.91 | - | 0.80 | - | 0.90 | - | 0.90 | - | 0.70 | - | 0.95 | - | 0.87 | - | 0.94 | - | 0.95 | - | 0.73 |
| *Epeorus assimilis* | EATON, 1885 | Ephemeroptera | - | X | - | 0.92 | - | 0.87 | - | 0.92 | - | 0.92 | - | 0.89 | - | 0.94 | - | 0.88 | - | 0.92 | - | 0.94 | - | 0.89 |
| *Ephemera danica* | MÜLLER, 1764 | Ephemeroptera | X | X | 0.98 | 0.85 | 0.92 | 0.83 | 0.93 | 0.84 | 0.82 | 0.85 | 0.66 | 0.84 | 0.97 | 0.88 | 0.92 | 0.87 | 0.93 | 0.87 | 0.84 | 0.88 | 0.68 | 0.86 |
| *Ephemerella mucronata* | BENGTSSON, 1909 | Ephemeroptera | - | X | - | 0.95 | - | 0.87 | - | 0.94 | - | 0.95 | - | 0.88 | - | 0.92 | - | 0.86 | - | 0.91 | - | 0.92 | - | 0.87 |
| *Habroleptoides confusa* | SARTORI & JACOB, 1986 | Ephemeroptera | - | X | - | 0.95 | - | 0.88 | - | 0.93 | - | 0.95 | - | 0.89 | - | 0.96 | - | 0.88 | - | 0.94 | - | 0.96 | - | 0.90 |
| *Habrophlebia lauta* | EATON, 1884 | Ephemeroptera | - | X | - | 0.87 | - | 0.84 | - | 0.87 | - | 0.90 | - | 0.86 | - | 0.92 | - | 0.87 | - | 0.90 | - | 0.92 | - | 0.88 |
| *Heptagenia sulphurea* | MÜLLER, 1776 | Ephemeroptera | X | X | 0.94 | 0.91 | 0.90 | 0.88 | 0.91 | 0.94 | 0.80 | 0.91 | 0.69 | 0.89 | 0.96 | 0.93 | 0.90 | 0.90 | 0.90 | 0.93 | 0.81 | 0.89 | 0.68 | 0.87 |
| *Leptophlebia submarginata* | LINNAEUS, 1767 | Ephemeroptera | X | - | 0.96 | - | 0.89 | - | 0.88 | - | 0.82 | - | 0.78 | - | 0.96 | - | 0.91 | - | 0.91 | - | 0.84 | - | 0.73 | - |
| *Paraleptophlebia submarginata* | STEPHENS, 1835 | Ephemeroptera | - | X | - | 0.84 | - | 0.82 | - | 0.84 | - | 0.87 | - | 0.82 | - | 0.87 | - | 0.84 | - | 0.88 | - | 0.91 | - | 0.86 |
| *Rhithrogena semicolorata* | CURTIS, 1834 | Ephemeroptera | - | X | - | 0.91 | - | 0.86 | - | 0.93 | - | 0.93 | - | 0.88 | - | 0.95 | - | 0.88 | - | 0.95 | - | 0.95 | - | 0.90 |
| *Serratella ignita* | PODA, 1761 | Ephemeroptera | - | X | - | 0.96 | - | 0.92 | - | 0.95 | - | 0.94 | - | 0.89 | - | 0.98 | - | 0.93 | - | 0.98 | - | 0.96 | - | 0.88 |
| *Torleya major* | KLAPÁLEK, 1905 | Ephemeroptera | - | X | - | 0.91 | - | 0.84 | - | 0.91 | - | 0.92 | - | 0.86 | - | 0.94 | - | 0.86 | - | 0.93 | - | 0.94 | - | 0.88 |
| *Ancylus fluviatilis* | O.F. MÜLLER, 1774 | Gastropoda | X | X | 0.94 | 0.88 | 0.92 | 0.84 | 0.85 | 0.87 | 0.78 | 0.85 | 0.67 | 0.83 | 0.97 | 0.91 | 0.94 | 0.88 | 0.93 | 0.91 | 0.86 | 0.90 | 0.73 | 0.88 |
| *Anisus vortex* | LINNAEUS, 1758 | Gastropoda | X | - | 0.94 | - | 0.87 | - | 0.96 | - | 0.88 | - | 0.76 | - | 0.95 | - | 0.91 | - | 0.94 | - | 0.89 | - | 0.77 | - |
| *Bithynia leachii* | SHEPPARD, 1823 | Gastropoda | X | - | 0.90 | - | 0.88 | - | 0.91 | - | 0.85 | - | 0.74 | - | 0.91 | - | 0.89 | - | 0.92 | - | 0.89 | - | 0.70 | - |
| *Bithynia tentaculata* | LINNAEUS, 1758 | Gastropoda | X | X | 0.97 | 0.95 | 0.91 | 0.89 | 0.95 | 0.93 | 0.90 | 0.92 | 0.78 | 0.88 | 0.97 | 0.96 | 0.91 | 0.90 | 0.93 | 0.93 | 0.90 | 0.93 | 0.73 | 0.86 |
| *Planorbarius corneus* | LINNAEUS, 1758 | Gastropoda | X | - | 0.85 | - | 0.90 | - | 0.96 | - | 0.91 | - | 0.75 | - | 0.89 | - | 0.92 | - | 0.96 | - | 0.90 | - | 0.71 | - |
| *Planorbis planorbis* | LINNAEUS, 1758 | Gastropoda | X | - | 0.95 | - | 0.85 | - | 0.94 | - | 0.92 | - | 0.67 | - | 0.93 | - | 0.90 | - | 0.92 | - | 0.92 | - | 0.67 | - |
| *Potamopyrgus antipodarum* | GRAY, 1843 | Gastropoda | - | X | - | 0.95 | - | 0.87 | - | 0.93 | - | 0.94 | - | 0.89 | - | 0.95 | - | 0.91 | - | 0.95 | - | 0.94 | - | 0.90 |
| *Radix balthica* | LINNAEUS, 1758 | Gastropoda | X | X | 0.94 | 0.84 | 0.89 | 0.82 | 0.94 | 0.85 | 0.83 | 0.84 | 0.53 | 0.82 | 0.90 | 0.87 | 0.90 | 0.85 | 0.92 | 0.87 | 0.85 | 0.89 | 0.59 | 0.85 |
| *Radix labiata* | ROSSMÄSSLER, 1835 | Gastropoda | - | X | - | 0.85 | - | 0.85 | - | 0.89 | - | 0.88 | - | 0.87 | - | 0.88 | - | 0.86 | - | 0.87 | - | 0.90 | - | 0.85 |
| *Aphelocheirus aestivalis* | FABRICIUS, 1794 | Heteroptera | - | X | - | 0.96 | - | 0.91 | - | 0.95 | - | 0.93 | - | 0.87 | - | 0.97 | - | 0.92 | - | 0.96 | - | 0.95 | - | 0.87 |
| *Erpobdella nigricollis* | BRANDES, 1900 | Hirudinea | X | X | 0.94 | 0.88 | 0.91 | 0.87 | 0.92 | 0.90 | 0.91 | 0.90 | 0.81 | 0.88 | 0.88 | 0.92 | 0.92 | 0.89 | 0.92 | 0.91 | 0.88 | 0.92 | 0.77 | 0.88 |
| *Erpobdella octoculata* | LINNAEUS, 1758 | Hirudinea | X | X | 0.92 | 0.86 | 0.89 | 0.85 | 0.92 | 0.88 | 0.88 | 0.85 | 0.70 | 0.84 | 0.94 | 0.91 | 0.92 | 0.89 | 0.94 | 0.91 | 0.88 | 0.91 | 0.69 | 0.89 |
| *Erpobdella vilnensis* | LISKIEWICZ, 1925 | Hirudinea | - | X | - | 0.89 | - | 0.85 | - | 0.89 | - | 0.87 | - | 0.86 | - | 0.89 | - | 0.88 | - | 0.88 | - | 0.90 | - | 0.89 |
| *Glossiphonia complanata* | LINNAEUS, 1758 | Hirudinea | X | X | 0.94 | 0.84 | 0.90 | 0.84 | 0.94 | 0.87 | 0.88 | 0.90 | 0.73 | 0.88 | 0.94 | 0.86 | 0.90 | 0.87 | 0.93 | 0.88 | 0.88 | 0.90 | 0.71 | 0.89 |
| *Glossiphonia nebulosa* | KALBE, 1964 | Hirudinea | X | X | 0.82 | 0.84 | 0.86 | 0.84 | 0.95 | 0.86 | 0.93 | 0.90 | 0.82 | 0.84 | 0.90 | 0.88 | 0.91 | 0.85 | 0.96 | 0.89 | 0.94 | 0.93 | 0.72 | 0.85 |
| *Helobdella stagnalis* | LINNAEUS, 1758 | Hirudinea | - | X | - | 0.88 | - | 0.84 | - | 0.86 | - | 0.87 | - | 0.86 | - | 0.91 | - | 0.87 | - | 0.89 | - | 0.89 | - | 0.87 |
| *Sialis fuliginosa* | PICTET, 1836 | Megaloptera | - | X | - | 0.75 | - | 0.72 | - | 0.81 | - | 0.81 | - | 0.78 | - | 0.86 | - | 0.83 | - | 0.88 | - | 0.89 | - | 0.84 |
| *Sialis lutaria* | LINNAEUS, 1758 | Megaloptera | X | X | 0.90 | 0.89 | 0.87 | 0.88 | 0.89 | 0.89 | 0.84 | 0.88 | 0.80 | 0.81 | 0.91 | 0.92 | 0.92 | 0.90 | 0.92 | 0.90 | 0.86 | 0.90 | 0.77 | 0.85 |
| *Calopteryx splendens* | HARRIS, 1782 | Odonata | X | X | 0.91 | 0.88 | 0.88 | 0.87 | 0.88 | 0.87 | 0.79 | 0.89 | 0.75 | 0.90 | 0.95 | 0.93 | 0.91 | 0.88 | 0.92 | 0.92 | 0.87 | 0.91 | 0.75 | 0.89 |
| *Calopteryx virgo* | LINNAEUS, 1758 | Odonata | - | X | - | 0.87 | - | 0.79 | - | 0.83 | - | 0.79 | - | 0.76 | - | 0.89 | - | 0.85 | - | 0.87 | - | 0.85 | - | 0.82 |
| *Eiseniella tetraedra* | SAVIGNY, 1826 | Oligochaeta | - | X | - | 0.80 | - | 0.79 | - | 0.81 | - | 0.82 | - | 0.81 | - | 0.87 | - | 0.86 | - | 0.89 | - | 0.89 | - | 0.88 |
| *Lumbriculus variegatus* | MÜLLER, 1774 | Oligochaeta | X | X | 0.90 | 0.87 | 0.82 | 0.87 | 0.94 | 0.89 | 0.86 | 0.85 | 0.79 | 0.85 | 0.91 | 0.90 | 0.90 | 0.90 | 0.92 | 0.90 | 0.86 | 0.91 | 0.76 | 0.90 |
| *Stylodrilus heringianus* | CLAPAREDE, 1862 | Oligochaeta | - | X | - | 0.92 | - | 0.87 | - | 0.93 | - | 0.94 | - | 0.90 | - | 0.93 | - | 0.88 | - | 0.93 | - | 0.93 | - | 0.90 |
| *Brachyptera risi* | MORTON, 1896 | Plecoptera | - | X | - | 0.88 | - | 0.83 | - | 0.87 | - | 0.89 | - | 0.85 | - | 0.94 | - | 0.87 | - | 0.93 | - | 0.93 | - | 0.89 |
| *Brachyptera seticornis* | KLAPALEK, 1902 | Plecoptera | - | X | - | 0.90 | - | 0.82 | - | 0.90 | - | 0.93 | - | 0.84 | - | 0.88 | - | 0.82 | - | 0.88 | - | 0.91 | - | 0.83 |
| *Isoperla grammatica* | PODA, 1761 | Plecoptera | X | - | 0.96 | - | 0.91 | - | 0.91 | - | 0.82 | - | 0.63 | - | 0.93 | - | 0.91 | - | 0.92 | - | 0.82 | - | 0.64 | - |
| *Leuctra hippopus* | KEMPNY, 1899 | Plecoptera | - | X | - | 0.85 | - | 0.85 | - | 0.87 | - | 0.88 | - | 0.85 | - | 0.91 | - | 0.86 | - | 0.87 | - | 0.88 | - | 0.85 |
| *Leuctra nigra* | OLIVIER, 1811 | Plecoptera | - | X | - | 0.97 | - | 0.91 | - | 0.95 | - | 0.96 | - | 0.94 | - | 0.94 | - | 0.88 | - | 0.92 | - | 0.92 | - | 0.91 |
| *Nemoura cinerea* | RETZIUS, 1783 | Plecoptera | X | - | 0.92 | - | 0.87 | - | 0.91 | - | 0.78 | - | 0.68 | - | 0.93 | - | 0.90 | - | 0.92 | - | 0.82 | - | 0.64 | - |
| *Siphonoperla torrentium* | PICTET, 1841 | Plecoptera | - | X | - | 0.92 | - | 0.90 | - | 0.96 | - | 0.95 | - | 0.91 | - | 0.90 | - | 0.87 | - | 0.93 | - | 0.92 | - | 0.89 |
| *Spongilla lacustris* | LINNAEUS, 1758 | Porifera | - | X | - | 0.92 | - | 0.86 | - | 0.85 | - | 0.85 | - | 0.81 | - | 0.96 | - | 0.84 | - | 0.88 | - | 0.87 | - | 0.87 |
| *Anabolia nervosa* | CURTIS, 1834 | Trichoptera | X | X | 0.96 | 0.82 | 0.88 | 0.80 | 0.93 | 0.82 | 0.84 | 0.87 | 0.75 | 0.84 | 0.95 | 0.91 | 0.90 | 0.88 | 0.92 | 0.89 | 0.86 | 0.91 | 0.80 | 0.87 |
| *Anomalopterygella chauviniana* | STEIN, 1874 | Trichoptera | - | X | - | 0.89 | - | 0.84 | - | 0.90 | - | 0.88 | - | 0.86 | - | 0.93 | - | 0.88 | - | 0.92 | - | 0.93 | - | 0.89 |
| *Athripsodes albifrons* | LINNAEUS, 1758 | Trichoptera | X | X | 0.97 | 0.88 | 0.90 | 0.81 | 0.93 | 0.85 | 0.85 | 0.88 | 0.78 | 0.87 | 0.94 | 0.89 | 0.92 | 0.83 | 0.93 | 0.87 | 0.90 | 0.88 | 0.78 | 0.87 |
| *Athripsodes bilineatus* | LINNAEUS, 1758 | Trichoptera | - | X | - | 0.91 | - | 0.90 | - | 0.86 | - | 0.89 | - | 0.88 | - | 0.91 | - | 0.86 | - | 0.87 | - | 0.89 | - | 0.86 |
| *Athripsodes cinereus* | CURTIS, 1834 | Trichoptera | X | X | 0.96 | 0.94 | 0.90 | 0.91 | 0.93 | 0.91 | 0.86 | 0.94 | 0.76 | 0.90 | 0.96 | 0.95 | 0.93 | 0.88 | 0.92 | 0.93 | 0.86 | 0.90 | 0.72 | 0.88 |
| *Brachycentrus subnubilus* | CURTIS, 1834 | Trichoptera | - | X | - | 0.97 | - | 0.92 | - | 0.95 | - | 0.94 | - | 0.88 | - | 0.97 | - | 0.92 | - | 0.96 | - | 0.95 | - | 0.86 |
| *Ceraclea albimacula* | RAMBUR, 1877 | Trichoptera | - | X | - | 0.96 | - | 0.93 | - | 0.92 | - | 0.92 | - | 0.90 | - | 0.95 | - | 0.91 | - | 0.91 | - | 0.87 | - | 0.87 |
| *Ceraclea dissimilis* | STEPHENS, 1836 | Trichoptera | - | X | - | 0.94 | - | 0.89 | - | 0.91 | - | 0.92 | - | 0.85 | - | 0.96 | - | 0.89 | - | 0.92 | - | 0.90 | - | 0.86 |
| *Chaetopteryx villosa* | FABRICIUS, 1789 | Trichoptera | - | X | - | 0.86 | - | 0.80 | - | 0.88 | - | 0.85 | - | 0.79 | - | 0.89 | - | 0.85 | - | 0.90 | - | 0.90 | - | 0.84 |
| *Cyrnus trimaculatus* | CURTIS, 1834 | Trichoptera | - | X | - | 0.92 | - | 0.87 | - | 0.89 | - | 0.89 | - | 0.81 | - | 0.95 | - | 0.87 | - | 0.92 | - | 0.92 | - | 0.82 |
| *Drusus annulatus* | STEPHENS, 1837 | Trichoptera | - | X | - | 0.90 | - | 0.87 | - | 0.86 | - | 0.95 | - | 0.87 | - | 0.89 | - | 0.84 | - | 0.84 | - | 0.92 | - | 0.83 |
| *Goera pilosa* | FABRICIUS, 1775 | Trichoptera | - | X | - | 0.89 | - | 0.87 | - | 0.90 | - | 0.92 | - | 0.85 | - | 0.88 | - | 0.87 | - | 0.88 | - | 0.89 | - | 0.87 |
| *Halesus digitatus* | SCHRANK, 1781 | Trichoptera | - | X | - | 0.85 | - | 0.83 | - | 0.90 | - | 0.90 | - | 0.81 | - | 0.87 | - | 0.85 | - | 0.92 | - | 0.91 | - | 0.86 |
| *Halesus radiatus* | CURTIS, 1834 | Trichoptera | X | X | 0.92 | 0.79 | 0.90 | 0.79 | 0.95 | 0.83 | 0.82 | 0.82 | 0.67 | 0.79 | 0.95 | 0.87 | 0.90 | 0.85 | 0.94 | 0.89 | 0.85 | 0.90 | 0.64 | 0.86 |
| *Hydropsyche angustipennis* | CURTIS, 1834 | Trichoptera | X | X | 0.97 | 0.93 | 0.91 | 0.89 | 0.95 | 0.94 | 0.83 | 0.93 | 0.70 | 0.92 | 0.93 | 0.94 | 0.91 | 0.89 | 0.93 | 0.94 | 0.84 | 0.93 | 0.69 | 0.92 |
| *Hydropsyche incognita* | PITSCH, 1993 | Trichoptera | - | X | - | 0.96 | - | 0.84 | - | 0.90 | - | 0.95 | - | 0.90 | - | 0.92 | - | 0.85 | - | 0.87 | - | 0.92 | - | 0.92 |
| *Hydropsyche instabilis* | CURTIS, 1834 | Trichoptera | - | X | - | 0.94 | - | 0.92 | - | 0.92 | - | 0.92 | - | 0.89 | - | 0.91 | - | 0.88 | - | 0.90 | - | 0.89 | - | 0.88 |
| *Hydropsyche pellucidula* | CURTIS, 1834 | Trichoptera | X | X | 0.95 | 0.81 | 0.91 | 0.79 | 0.94 | 0.82 | 0.83 | 0.81 | 0.68 | 0.82 | 0.95 | 0.90 | 0.91 | 0.88 | 0.93 | 0.90 | 0.84 | 0.89 | 0.66 | 0.89 |
| *Hydropsyche saxonica* | McLACHLAN, 1884 | Trichoptera | - | X | - | 0.87 | - | 0.83 | - | 0.87 | - | 0.86 | - | 0.84 | - | 0.90 | - | 0.85 | - | 0.91 | - | 0.89 | - | 0.86 |
| *Hydropsyche siltalai* | DÖHLER, 1963 | Trichoptera | X | X | 0.96 | 0.91 | 0.93 | 0.87 | 0.93 | 0.90 | 0.83 | 0.91 | 0.72 | 0.88 | 0.96 | 0.94 | 0.91 | 0.89 | 0.91 | 0.93 | 0.85 | 0.93 | 0.72 | 0.91 |
| *Lepidostoma basale* | F. KOLENATI, 1848 | Trichoptera | - | X | - | 0.89 | - | 0.82 | - | 0.89 | - | 0.91 | - | 0.86 | - | 0.94 | - | 0.83 | - | 0.92 | - | 0.94 | - | 0.88 |
| *Lepidostoma hirtum* | FABRICIUS, 1775 | Trichoptera | X | X | 0.97 | 0.89 | 0.93 | 0.84 | 0.84 | 0.89 | 0.84 | 0.90 | 0.86 | 0.91 | 0.97 | 0.90 | 0.92 | 0.86 | 0.92 | 0.90 | 0.84 | 0.92 | 0.81 | 0.89 |
| *Limnephilus lunatus* | CURTIS, 1834 | Trichoptera | X | - | 0.94 | - | 0.86 | - | 0.92 | - | 0.83 | - | 0.56 | - | 0.94 | - | 0.90 | - | 0.90 | - | 0.82 | - | 0.61 | - |
| *Lype phaeopa* | J.F. STEPHENS, 1836 | Trichoptera | - | X | - | 0.94 | - | 0.85 | - | 0.89 | - | 0.89 | - | 0.84 | - | 0.91 | - | 0.84 | - | 0.88 | - | 0.90 | - | 0.85 |
| *Lype reducta* | HAGEN, 1868 | Trichoptera | X | X | 0.95 | 0.82 | 0.86 | 0.85 | 0.85 | 0.83 | 0.88 | 0.83 | 0.84 | 0.81 | 0.97 | 0.81 | 0.93 | 0.85 | 0.89 | 0.88 | 0.85 | 0.82 | 0.77 | 0.84 |
| *Micrasema longulum* | McLACHLAN, 1876 | Trichoptera | - | X | - | 0.84 | - | 0.85 | - | 0.85 | - | 0.91 | - | 0.79 | - | 0.88 | - | 0.84 | - | 0.86 | - | 0.91 | - | 0.79 |
| *Mystacides azurea* | LINNAEUS, 1761 | Trichoptera | - | X | - | 0.91 | - | 0.83 | - | 0.91 | - | 0.90 | - | 0.83 | - | 0.88 | - | 0.83 | - | 0.89 | - | 0.89 | - | 0.79 |
| *Mystacides nigra* | LINNAEUS, 1758 | Trichoptera | - | X | - | 0.92 | - | 0.85 | - | 0.92 | - | 0.90 | - | 0.85 | - | 0.90 | - | 0.86 | - | 0.92 | - | 0.88 | - | 0.86 |
| *Neureclipsis bimaculata* | LINNAEUS, 1758 | Trichoptera | - | X | - | 0.99 | - | 0.87 | - | 0.96 | - | 0.96 | - | 0.90 | - | 0.97 | - | 0.86 | - | 0.92 | - | 0.92 | - | 0.85 |
| *Odontocerum albicorne* | SCOPOLI, 1763 | Trichoptera | - | X | - | 0.91 | - | 0.89 | - | 0.92 | - | 0.93 | - | 0.86 | - | 0.90 | - | 0.87 | - | 0.90 | - | 0.92 | - | 0.85 |
| *Philopotamus montanus* | E. DONOVAN, 1813 | Trichoptera | - | X | - | 0.94 | - | 0.83 | - | 0.89 | - | 0.91 | - | 0.84 | - | 0.90 | - | 0.82 | - | 0.85 | - | 0.89 | - | 0.86 |
| *Plectrocnemia conspersa* | CURTIS, 1834 | Trichoptera | - | X | - | 0.85 | - | 0.85 | - | 0.88 | - | 0.90 | - | 0.87 | - | 0.88 | - | 0.87 | - | 0.88 | - | 0.90 | - | 0.88 |
| *Polycentropus flavomaculatus* | PICTET, 1834 | Trichoptera | - | X | - | 0.86 | - | 0.82 | - | 0.88 | - | 0.88 | - | 0.87 | - | 0.89 | - | 0.84 | - | 0.89 | - | 0.89 | - | 0.87 |
| *Polycentropus irroratus* | CURTIS, 1835 | Trichoptera | X | X | 0.94 | 0.95 | 0.86 | 0.86 | 0.90 | 0.95 | 0.82 | 0.93 | 0.71 | 0.90 | 0.95 | 0.94 | 0.91 | 0.88 | 0.95 | 0.94 | 0.83 | 0.91 | 0.67 | 0.88 |
| *Potamophylax cingulatus* | STEPHENS, 1837 | Trichoptera | X | X | 0.95 | 0.80 | 0.87 | 0.82 | 0.94 | 0.81 | 0.87 | 0.86 | 0.70 | 0.82 | 0.95 | 0.89 | 0.92 | 0.86 | 0.92 | 0.88 | 0.83 | 0.91 | 0.65 | 0.84 |
| *Potamophylax latipennis* | CURTIS, 1834 | Trichoptera | X | X | 0.95 | 0.81 | 0.88 | 0.84 | 0.95 | 0.85 | 0.87 | 0.86 | 0.70 | 0.84 | 0.92 | 0.86 | 0.92 | 0.87 | 0.91 | 0.87 | 0.84 | 0.89 | 0.68 | 0.85 |
| *Potamophylax luctuosus* | PILLER & MITTERPACHER, 1783 | Trichoptera | X | X | 0.94 | 0.81 | 0.87 | 0.84 | 0.92 | 0.84 | 0.84 | 0.86 | 0.70 | 0.85 | 0.90 | 0.88 | 0.90 | 0.85 | 0.91 | 0.87 | 0.85 | 0.89 | 0.70 | 0.84 |
| *Potamophylax rotundipennis* | BRAUER, 1857 | Trichoptera | - | X | - | 0.88 | - | 0.86 | - | 0.88 | - | 0.92 | - | 0.90 | - | 0.90 | - | 0.83 | - | 0.89 | - | 0.85 | - | 0.85 |
| *Psychomyia pusilla* | FABRICIUS, 1781 | Trichoptera | - | X | - | 0.93 | - | 0.91 | - | 0.93 | - | 0.93 | - | 0.87 | - | 0.94 | - | 0.90 | - | 0.93 | - | 0.94 | - | 0.86 |
| *Rhyacophila dorsalis* | CURTIS, 1834 | Trichoptera | - | X | - | 0.89 | - | 0.80 | - | 0.87 | - | 0.90 | - | 0.84 | - | 0.90 | - | 0.83 | - | 0.90 | - | 0.93 | - | 0.86 |
| *Rhyacophila evoluta* | McLACHLAN, 1879 | Trichoptera | - | X | - | 0.84 | - | 0.78 | - | 0.85 | - | 0.83 | - | 0.81 | - | 0.89 | - | 0.83 | - | 0.89 | - | 0.90 | - | 0.83 |
| *Rhyacophila fasciata* | HAGEN, 1859 | Trichoptera | - | X | - | 0.88 | - | 0.83 | - | 0.90 | - | 0.91 | - | 0.85 | - | 0.87 | - | 0.81 | - | 0.84 | - | 0.88 | - | 0.89 |
| *Rhyacophila nubila* | ZETTERSTEDT, 1840 | Trichoptera | - | X | - | 0.91 | - | 0.86 | - | 0.91 | - | 0.86 | - | 0.83 | - | 0.85 | - | 0.79 | - | 0.86 | - | 0.78 | - | 0.81 |
| *Rhyacophila obliterata* | McLACHLAN, 1863 | Trichoptera | - | X | - | 0.89 | - | 0.78 | - | 0.91 | - | 0.90 | - | 0.87 | - | 0.89 | - | 0.80 | - | 0.91 | - | 0.88 | - | 0.85 |
| *Rhyacophila tristis* | PICTET, 1834 | Trichoptera | - | X | - | 0.86 | - | 0.76 | - | 0.85 | - | 0.87 | - | 0.79 | - | 0.86 | - | 0.78 | - | 0.87 | - | 0.88 | - | 0.83 |
| *Sericostoma flavicorne* | SCHNEIDER, 1845 | Trichoptera | - | X | - | 0.93 | - | 0.90 | - | 0.92 | - | 0.94 | - | 0.92 | - | 0.86 | - | 0.89 | - | 0.90 | - | 0.86 | - | 0.90 |
| *Sericostoma personatum* | KIRBY & SPENCER, 1826 | Trichoptera | - | X | - | 0.90 | - | 0.85 | - | 0.90 | - | 0.94 | - | 0.85 | - | 0.89 | - | 0.85 | - | 0.90 | - | 0.93 | - | 0.86 |
| *Silo nigricornis* | PICTET, 1834 | Trichoptera | - | X | - | 0.89 | - | 0.86 | - | 0.92 | - | 0.90 | - | 0.88 | - | 0.86 | - | 0.80 | - | 0.85 | - | 0.82 | - | 0.88 |
| *Silo pallipes* | FABRICIUS, 1781 | Trichoptera | - | X | - | 0.87 | - | 0.87 | - | 0.87 | - | 0.90 | - | 0.82 | - | 0.88 | - | 0.88 | - | 0.87 | - | 0.92 | - | 0.86 |
| *Silo piceus* | BRAUER, 1857 | Trichoptera | - | X | - | 0.90 | - | 0.86 | - | 0.91 | - | 0.87 | - | 0.86 | - | 0.89 | - | 0.84 | - | 0.87 | - | 0.86 | - | 0.84 |
| *Dendrocoelum lacteum* | O.F. MÜLLER, 1774 | Turbellaria | - | X | - | 0.88 | - | 0.87 | - | 0.91 | - | 0.84 | - | 0.84 | - | 0.91 | - | 0.90 | - | 0.92 | - | 0.88 | - | 0.90 |
| *Dugesia gonocephala* | DUGES, 1830 | Turbellaria | X | X | 0.96 | 0.89 | 0.93 | 0.86 | 0.91 | 0.90 | 0.86 | 0.89 | 0.68 | 0.86 | 0.94 | 0.93 | 0.94 | 0.89 | 0.93 | 0.92 | 0.85 | 0.93 | 0.69 | 0.90 |
| *Dugesia lugubris* | SCHMIDT, 1861 | Turbellaria | - | X | - | 0.90 | - | 0.87 | - | 0.86 | - | 0.92 | - | 0.87 | - | 0.92 | - | 0.88 | - | 0.90 | - | 0.92 | - | 0.88 |
| *Dugesia polychroa* | SCHMIDT, 1861 | Turbellaria | - | X | - | 0.90 | - | 0.87 | - | 0.87 | - | 0.92 | - | 0.86 | - | 0.93 | - | 0.89 | - | 0.91 | - | 0.91 | - | 0.88 |
| *Polycelis felina* | DALYELL, 1814 | Turbellaria | - | X | - | 0.91 | - | 0.87 | - | 0.90 | - | 0.91 | - | 0.83 | - | 0.90 | - | 0.85 | - | 0.90 | - | 0.88 | - | 0.86 |
| *Polycelis nigra* | MUELLER, 1774 | Turbellaria | - | X | - | 0.91 | - | 0.88 | - | 0.83 | - | 0.89 | - | 0.82 | - | 0.92 | - | 0.89 | - | 0.89 | - | 0.89 | - | 0.83 |
| *Polycelis tenuis* | IJIMA, 1884 | Turbellaria | - | X | - | 0.90 | - | 0.87 | - | 0.83 | - | 0.87 | - | 0.84 | - | 0.90 | - | 0.88 | - | 0.86 | - | 0.90 | - | 0.86 |

**Table S2.** Mean species assemblage responses (SARs) to five IHA metrics and overall species assemblage responses (OSARs) according to the weighted mean aggregation method in two projected periods of Horizons 2050 and 2090 in the Kinzig river catchment.

| Site | Stream order | Horizon 2050 | | | | | | Horizon 2090 | | | | | |  |
| --- | --- | --- | --- | --- | --- | --- | --- | --- | --- | --- | --- | --- | --- | --- |
|  |  | Duration | Frequency | Magnitude | Rate | Timing | Weighted mean | Duration | Frequency | Magnitude | Rate | Timing | Weighted mean | |
| 1 | 3 | 69.36 | 23.48 | -12.64 | -7.01 | 33.82 | 37.24 | 52.98 | -1.29 | -9.01 | 0.08 | 25.85 | 25.05 | |
| 2 | 3 | 37.20 | 13.33 | 0.54 | 11.14 | 5.73 | 17.52 | 19.43 | 2.84 | -11.53 | 8.47 | 11.14 | 6.07 | |
| 3 | 1 | -0.72 | 13.33 | 0.54 | 4.16 | 5.73 | 4.61 | -0.23 | 2.84 | -11.53 | 4.82 | 11.14 | 1.41 | |
| 4 | 3 | 20.00 | 2.54 | 17.16 | -0.88 | 11.76 | 11.76 | 13.58 | 6.85 | 2.32 | 3.01 | 21.65 | 11.51 | |
| 5 | 3 | 19.66 | 2.54 | 17.16 | -2.90 | 11.76 | 9.65 | 13.46 | 6.85 | 2.32 | 4.94 | 21.65 | 11.81 | |
| 6 | 3 | 13.48 | -1.98 | 19.72 | 3.47 | 23.52 | 13.62 | 10.87 | 5.83 | 5.28 | 14.95 | 28.59 | 15.68 | |
| 7 | 3 | 12.88 | -1.98 | 19.72 | 1.02 | 23.52 | 13.11 | 10.46 | 5.83 | 5.28 | 9.46 | 28.59 | 14.70 | |
| 8 | 3 | 6.90 | -1.98 | 19.72 | 0.04 | 23.52 | 11.95 | 6.28 | 5.83 | 5.28 | 12.19 | 28.59 | 14.46 | |
| 9 | 3 | 6.90 | -1.98 | 19.72 | 1.06 | 23.52 | 12.12 | 6.28 | 5.83 | 5.28 | 13.03 | 28.59 | 14.60 | |
| 10 | 2 | 0.93 | -1.98 | 19.72 | 0.49 | 23.52 | 11.03 | 0.93 | 5.83 | 5.28 | 16.76 | 28.59 | 14.33 | |
| 11 | 2 | 0.42 | -1.98 | 19.72 | -5.21 | 23.52 | 10.00 | 0.26 | 5.83 | 5.28 | 14.36 | 28.59 | 13.82 | |
| 12 | 1 | -0.83 | -1.98 | 19.72 | 6.67 | 23.52 | 11.77 | -0.52 | 5.83 | 5.28 | 13.01 | 28.59 | 13.47 | |
| 13 | 1 | -0.83 | -1.98 | 19.72 | 6.67 | 23.52 | 11.77 | -0.52 | 5.83 | 5.28 | 13.01 | 28.59 | 13.47 | |
| 14 | 1 | -0.83 | -1.98 | 19.72 | 6.67 | 23.52 | 11.77 | -0.52 | 5.83 | 5.28 | 13.01 | 28.59 | 13.47 | |
| 15 | 1 | -0.89 | -1.98 | 19.72 | 13.27 | 23.52 | 12.86 | -0.50 | 5.83 | 5.28 | 5.82 | 28.59 | 12.27 | |
| 16 | 1 | -0.89 | -1.98 | 19.72 | 13.27 | 23.52 | 12.86 | -0.50 | 5.83 | 5.28 | 5.82 | 28.59 | 12.27 | |
| 17 | 1 | -0.89 | -1.98 | 19.72 | 13.27 | 23.52 | 12.86 | -0.50 | 5.83 | 5.28 | 5.82 | 28.59 | 12.27 | |
| 18 | 1 | -0.89 | -1.98 | 19.72 | 12.25 | 23.52 | 12.69 | -0.56 | 5.83 | 5.28 | 2.45 | 28.59 | 11.70 | |
| 19 | 1 | -0.89 | -1.98 | 19.72 | 12.25 | 23.52 | 12.69 | -0.56 | 5.83 | 5.28 | 2.45 | 28.59 | 11.70 | |
| 20 | 1 | -0.89 | -1.98 | 19.72 | 12.25 | 23.52 | 12.69 | -0.56 | 5.83 | 5.28 | 2.45 | 28.59 | 11.70 | |
| 21 | 1 | -0.94 | -1.98 | 19.72 | -8.37 | 23.52 | 9.24 | -0.54 | 5.83 | 5.28 | 8.87 | 28.59 | 12.77 | |
| 22 | 2 | 0.47 | -1.98 | 19.72 | -5.21 | 23.52 | 10.01 | 0.33 | 5.83 | 5.28 | 11.71 | 28.59 | 13.39 | |
| 23 | 2 | 0.51 | -1.98 | 19.72 | -5.77 | 23.52 | 9.92 | 0.33 | 5.83 | 5.28 | 11.65 | 28.59 | 13.38 | |
| 24 | 2 | 0.45 | -1.98 | 19.72 | -6.85 | 23.52 | 9.73 | 0.31 | 5.83 | 5.28 | 11.33 | 28.59 | 13.32 | |
| 25 | 2 | 0.50 | -1.98 | 19.72 | -4.85 | 23.52 | 10.07 | 0.34 | 5.83 | 5.28 | 11.78 | 28.59 | 13.40 | |
| 26 | 1 | -0.90 | -1.98 | 19.72 | 14.69 | 23.52 | 13.10 | -0.53 | 5.83 | 5.28 | 13.58 | 28.59 | 13.56 | |
| 27 | 1 | -0.90 | -1.98 | 19.72 | 14.69 | 23.52 | 13.10 | -0.53 | 5.83 | 5.28 | 13.58 | 28.59 | 13.56 | |
| 28 | 1 | -0.85 | -1.98 | 19.72 | 11.57 | 23.52 | 12.58 | -0.52 | 5.83 | 5.28 | 8.93 | 28.59 | 12.78 | |
| 29 | 2 | 5.99 | 7.04 | -3.95 | 0.67 | 7.97 | 3.54 | 6.40 | 7.64 | 1.79 | 22.81 | 25.73 | 16.13 | |
| 30 | 2 | 13.54 | -2.66 | 16.28 | 6.13 | 1.12 | 6.88 | 10.29 | 2.98 | 1.34 | 10.36 | 17.75 | 8.54 | |
| 31 | 2 | -0.31 | 9.19 | -16.90 | 16.98 | 6.34 | 3.06 | 0.13 | 1.42 | -8.95 | -1.05 | 21.14 | 5.64 | |
| 32 | 2 | -0.31 | 9.19 | -16.90 | 16.98 | 6.34 | 3.06 | 0.13 | 1.42 | -8.95 | -1.05 | 21.14 | 5.64 | |
| 33 | 2 | 20.33 | -1.64 | -4.01 | 5.79 | 19.61 | 10.07 | 9.47 | 6.00 | -10.31 | 5.18 | 41.24 | 19.15 | |
| 34 | 2 | 1.02 | 23.75 | 0.23 | -3.53 | 20.91 | 12.43 | 1.02 | -3.03 | -11.48 | 8.41 | 31.48 | 9.65 | |
| 35 | 1 | -0.76 | 23.75 | 0.23 | 1.82 | 20.91 | 12.94 | -0.16 | -3.03 | -11.48 | 17.01 | 31.48 | 10.88 | |
| 36 | 1 | -0.86 | 12.22 | -18.91 | 6.62 | 37.76 | 12.43 | -0.17 | 0.58 | -15.17 | 3.03 | 33.09 | 9.07 | |
| 37 | 1 | -0.47 | 11.58 | -17.34 | -9.08 | 34.66 | 9.00 | -0.15 | -8.09 | -13.36 | -14.18 | 30.10 | 4.07 | |
| 38 | 1 | -0.49 | 11.58 | -17.34 | -15.99 | 34.66 | 7.85 | -0.11 | -8.09 | -13.36 | -8.12 | 30.10 | 5.09 | |
| 39 | 1 | -0.40 | 11.58 | -17.34 | -8.80 | 34.66 | 9.06 | -0.12 | -8.09 | -13.36 | -14.57 | 30.10 | 4.01 | |
| 40 | 1 | -0.47 | 11.58 | -17.34 | -4.22 | 34.66 | 9.81 | -0.12 | -8.09 | -13.36 | -4.08 | 30.10 | 5.76 | |
| 41 | 1 | -0.57 | 11.58 | -17.34 | -13.16 | 34.66 | 8.31 | -0.17 | -8.09 | -13.36 | -3.25 | 30.10 | 5.89 | |
| 42 | 1 | -0.61 | 11.58 | -17.34 | -5.11 | 34.66 | 9.64 | -0.13 | -8.09 | -13.36 | 5.47 | 30.10 | 7.35 | |
| 43 | 1 | -0.60 | 11.58 | -17.34 | -5.39 | 34.66 | 9.60 | -0.12 | -8.09 | -13.36 | 6.62 | 30.10 | 7.54 | |
| 44 | 2 | 9.66 | 7.50 | 4.02 | -8.27 | 30.45 | 12.30 | 3.06 | 9.40 | 8.96 | -2.69 | 22.72 | 10.69 | |
| 45 | 2 | 1.23 | 7.50 | 4.02 | -9.23 | 30.45 | 10.74 | -0.90 | 9.40 | 8.96 | -0.55 | 22.72 | 10.39 | |
| 46 | 2 | 5.12 | 7.50 | 4.02 | -4.20 | 30.45 | 12.22 | 1.74 | 9.40 | 8.96 | 3.74 | 22.72 | 11.55 | |
| 47 | 1 | -1.04 | 2.03 | -3.33 | 8.48 | 35.15 | 12.74 | -0.38 | 12.59 | -1.02 | -5.66 | 27.95 | 10.24 | |
| 48 | 1 | -1.05 | 2.03 | -3.33 | -6.05 | 35.15 | 10.32 | -0.38 | 12.59 | -1.02 | 4.89 | 27.95 | 12.00 | |
| 49 | 1 | -0.94 | 2.03 | -3.33 | -6.99 | 35.15 | 10.18 | -0.30 | 12.59 | -1.02 | -2.53 | 27.95 | 10.77 | |
| 50 | 1 | -0.89 | 2.03 | -3.33 | 5.58 | 35.15 | 12.28 | -0.25 | 12.59 | -1.02 | 1.31 | 27.95 | 11.42 | |
| 51 | 1 | -0.76 | 18.23 | -13.10 | -14.30 | 33.82 | 9.62 | -0.12 | -8.93 | -8.93 | 10.54 | 33.55 | 9.94 | |
| 52 | 1 | -0.76 | 18.23 | -13.10 | -13.15 | 33.82 | 9.81 | -0.15 | -8.93 | -8.93 | 14.55 | 33.55 | 10.61 | |
| 53 | 1 | -0.76 | 18.23 | -13.10 | -13.15 | 33.82 | 9.81 | -0.15 | -8.93 | -8.93 | 14.55 | 33.55 | 10.61 | |
| 54 | 1 | -0.89 | 18.23 | -13.10 | 8.03 | 33.82 | 13.32 | -0.16 | -8.93 | -8.93 | -2.20 | 33.55 | 7.81 | |
| 55 | 1 | -0.87 | 18.23 | -13.10 | 17.19 | 36.01 | 15.58 | -0.19 | -8.93 | -8.93 | 16.00 | 33.55 | 10.84 | |
| 56 | 1 | -0.80 | 18.23 | -13.10 | -6.35 | 33.82 | 10.94 | -0.18 | -8.93 | -8.93 | 19.72 | 33.55 | 11.46 | |
| 57 | 1 | -0.85 | 18.23 | -13.10 | 17.01 | 33.82 | 14.82 | -0.13 | -8.93 | -8.93 | 16.27 | 33.55 | 10.90 | |
| 58 | 1 | -0.79 | 18.23 | -13.10 | -7.55 | 33.82 | 10.74 | -0.13 | -8.93 | -8.93 | 21.85 | 33.55 | 13.26 | |
| 59 | 1 | -0.79 | 18.23 | -13.10 | -8.65 | 33.82 | 10.55 | -0.13 | -8.93 | -8.93 | 21.46 | 33.55 | 13.15 | |
| 60 | 1 | -0.77 | 18.23 | -13.10 | -15.57 | 33.82 | 9.40 | -0.14 | -8.93 | -8.93 | 8.79 | 33.55 | 9.65 | |
| 61 | 1 | -0.78 | 18.23 | -13.10 | -14.57 | 33.82 | 9.57 | -0.17 | -8.93 | -8.93 | 9.70 | 33.55 | 9.79 | |
| 62 | 1 | -0.78 | 18.23 | -13.10 | -14.57 | 33.82 | 9.57 | -0.17 | -8.93 | -8.93 | 9.70 | 33.55 | 9.79 | |
| 63 | 1 | -0.78 | 18.23 | -13.10 | -14.57 | 33.82 | 9.57 | -0.17 | -8.93 | -8.93 | 9.70 | 33.55 | 9.79 | |
| 64 | 3 | 67.03 | 18.23 | -13.10 | -7.97 | 33.82 | 36.99 | 53.80 | -8.93 | -8.93 | 0.60 | 33.55 | 26.40 | |
| 65 | 3 | 66.96 | 18.23 | -13.10 | -8.03 | 33.82 | 36.95 | 53.71 | -8.93 | -8.93 | 0.73 | 33.55 | 26.39 | |
| 66 | 3 | 60.42 | 11.58 | -17.34 | -7.11 | 34.66 | 33.12 | 47.79 | -8.09 | -13.36 | 3.03 | 30.10 | 23.14 | |
| 67 | 3 | 60.41 | 11.58 | -17.34 | -7.12 | 34.66 | 33.12 | 47.78 | -8.09 | -13.36 | 3.13 | 30.10 | 23.15 | |
| 68 | 3 | 60.42 | 11.58 | -17.34 | -7.12 | 34.66 | 33.12 | 47.76 | -8.09 | -13.36 | 3.15 | 30.10 | 23.15 | |
| 69 | 3 | 60.42 | 11.58 | -17.34 | -7.12 | 34.66 | 33.12 | 47.76 | -8.09 | -13.36 | 3.15 | 30.10 | 23.15 | |
| 70 | 3 | 60.46 | 11.58 | -17.34 | -6.57 | 34.66 | 33.20 | 47.78 | -8.09 | -13.36 | 3.22 | 30.10 | 23.16 | |
| 71 | 3 | 60.46 | 11.58 | -17.34 | -6.57 | 34.66 | 33.21 | 47.77 | -8.09 | -13.36 | 3.23 | 30.10 | 23.16 | |
| 72 | 3 | 60.46 | 11.58 | -17.34 | -6.57 | 34.66 | 33.21 | 47.77 | -8.09 | -13.36 | 3.23 | 30.10 | 23.16 | |
| 73 | 3 | 60.46 | 11.58 | -17.34 | -6.57 | 34.66 | 33.21 | 47.77 | -8.09 | -13.36 | 3.23 | 30.10 | 23.16 | |
| 74 | 3 | 61.06 | 11.58 | -17.34 | -6.60 | 34.66 | 33.47 | 49.11 | -8.09 | -13.36 | 2.36 | 30.10 | 23.55 | |
| 75 | 3 | 61.06 | 11.58 | -17.34 | -6.60 | 34.66 | 33.47 | 49.11 | -8.09 | -13.36 | 2.36 | 30.10 | 23.55 | |
| 76 | 3 | 61.06 | 11.58 | -17.34 | -6.60 | 34.66 | 33.47 | 49.11 | -8.09 | -13.36 | 2.36 | 30.10 | 23.55 | |
| 77 | 3 | 62.32 | 11.58 | -17.34 | -6.93 | 34.66 | 33.99 | 53.70 | -8.09 | -13.36 | 2.67 | 30.10 | 25.31 | |
| 78 | 3 | 62.32 | 11.58 | -17.34 | -6.93 | 34.66 | 33.99 | 53.70 | -8.09 | -13.36 | 2.67 | 30.10 | 25.31 | |
| 79 | 3 | 62.32 | 11.58 | -17.34 | -6.93 | 34.66 | 33.99 | 53.70 | -8.09 | -13.36 | 2.67 | 30.10 | 25.31 | |
| 80 | 3 | 69.17 | 23.48 | -12.64 | -7.40 | 33.82 | 37.12 | 53.01 | -1.29 | -9.01 | -0.41 | 25.85 | 25.00 | |
| 81 | 3 | 69.01 | 23.48 | -12.64 | -7.30 | 33.82 | 37.07 | 53.00 | -1.29 | -9.01 | -0.47 | 25.85 | 24.99 | |
| 82 | 3 | 62.29 | 12.22 | -18.91 | 1.16 | 37.76 | 35.46 | 52.73 | 0.58 | -15.17 | 2.33 | 33.09 | 26.51 | |
| 83 | 3 | 62.29 | 12.22 | -18.91 | 1.16 | 37.76 | 35.46 | 52.73 | 0.58 | -15.17 | 2.33 | 33.09 | 26.51 | |
| 84 | 3 | 62.29 | 12.22 | -18.91 | 1.16 | 37.76 | 35.46 | 52.73 | 0.58 | -15.17 | 2.33 | 33.09 | 26.51 | |
| 85 | 3 | 62.29 | 12.22 | -18.91 | 1.16 | 37.76 | 35.46 | 52.73 | 0.58 | -15.17 | 2.33 | 33.09 | 26.51 | |
| 86 | 3 | 62.36 | 12.22 | -18.91 | 1.29 | 37.76 | 35.51 | 53.08 | 0.58 | -15.17 | 3.54 | 33.09 | 26.80 | |
| 87 | 3 | 62.36 | 12.22 | -18.91 | 1.29 | 37.76 | 35.51 | 53.08 | 0.58 | -15.17 | 3.54 | 33.09 | 26.80 | |
| 88 | 3 | 62.36 | 12.22 | -18.91 | 1.29 | 37.76 | 35.51 | 53.08 | 0.58 | -15.17 | 3.54 | 33.09 | 26.80 | |
| 89 | 3 | 62.37 | 12.22 | -18.91 | 0.65 | 37.76 | 35.44 | 53.33 | 0.58 | -15.17 | 3.48 | 33.09 | 26.88 | |
| 90 | 3 | 62.37 | 12.22 | -18.91 | 0.65 | 37.76 | 35.44 | 53.33 | 0.58 | -15.17 | 3.48 | 33.09 | 26.88 | |
| 91 | 3 | 62.37 | 12.22 | -18.91 | 0.65 | 37.76 | 35.44 | 53.33 | 0.58 | -15.17 | 3.48 | 33.09 | 26.88 | |
| 92 | 3 | 62.37 | 12.22 | -18.91 | 0.65 | 37.76 | 35.44 | 53.33 | 0.58 | -15.17 | 3.48 | 33.09 | 26.88 | |
| 93 | 3 | 62.46 | 12.22 | -18.91 | 0.92 | 37.76 | 35.51 | 54.08 | 0.58 | -15.17 | 3.02 | 33.09 | 27.10 | |
| 94 | 3 | 62.46 | 12.22 | -18.91 | 0.92 | 37.76 | 35.51 | 54.08 | 0.58 | -15.17 | 3.02 | 33.09 | 27.10 | |
| 95 | 3 | 62.77 | 4.48 | -20.84 | -3.48 | 36.61 | 28.36 | 54.51 | 4.25 | -17.79 | 15.75 | 37.13 | 30.00 | |
| 96 | 3 | 62.83 | 4.48 | -20.84 | -3.23 | 36.61 | 28.41 | 54.53 | 4.25 | -17.79 | 15.48 | 37.13 | 29.97 | |
| 97 | 3 | 62.83 | 4.48 | -20.84 | -3.23 | 36.61 | 28.41 | 54.53 | 4.25 | -17.79 | 15.48 | 37.13 | 29.97 | |
| 98 | 3 | 62.83 | 4.48 | -20.84 | -3.23 | 36.61 | 28.41 | 54.53 | 4.25 | -17.79 | 15.48 | 37.13 | 29.97 | |
| 99 | 3 | 62.74 | 4.48 | -20.84 | -3.15 | 36.61 | 28.38 | 54.55 | 4.25 | -17.79 | 16.03 | 37.13 | 30.05 | |
| 100 | 3 | 62.65 | 4.48 | -20.84 | -2.99 | 36.61 | 28.36 | 54.42 | 4.25 | -17.79 | 15.80 | 37.13 | 29.97 | |
| 101 | 3 | 62.65 | 4.48 | -20.84 | -2.99 | 36.61 | 28.36 | 54.42 | 4.25 | -17.79 | 15.80 | 37.13 | 29.97 | |
| 102 | 3 | 62.65 | 4.48 | -20.84 | -2.99 | 36.61 | 28.36 | 54.42 | 4.25 | -17.79 | 15.80 | 37.13 | 29.97 | |
| 103 | 3 | 62.55 | 4.48 | -20.84 | -2.48 | 36.61 | 28.37 | 53.76 | 4.25 | -17.79 | 15.28 | 37.13 | 29.66 | |
| 104 | 3 | 62.60 | 4.48 | -20.84 | -3.20 | 36.61 | 28.32 | 53.66 | 4.25 | -17.79 | 15.99 | 37.13 | 29.71 | |
| 105 | 3 | 62.52 | 4.48 | -20.84 | -3.61 | 36.61 | 28.25 | 53.65 | 4.25 | -17.79 | 15.43 | 37.13 | 29.64 | |
| 106 | 3 | 62.57 | 4.48 | -20.84 | -3.31 | 36.61 | 28.30 | 53.66 | 4.25 | -17.79 | 16.01 | 37.13 | 29.71 | |
| 107 | 3 | 62.53 | 4.48 | -20.84 | -3.72 | 36.61 | 28.24 | 53.68 | 4.25 | -17.79 | 15.37 | 37.13 | 29.64 | |
| 108 | 3 | 62.83 | 4.48 | -20.84 | -3.34 | 36.61 | 28.40 | 53.63 | 4.25 | -17.79 | 15.06 | 37.13 | 29.58 | |
| 109 | 3 | 62.95 | 4.48 | -20.84 | -2.82 | 36.61 | 28.50 | 53.63 | 4.25 | -17.79 | 15.28 | 37.13 | 29.61 | |
| 110 | 3 | 62.93 | 4.48 | -20.84 | -2.83 | 36.61 | 28.49 | 53.58 | 4.25 | -17.79 | 15.26 | 37.13 | 29.59 | |
| 111 | 2 | 21.34 | 3.23 | -21.20 | 5.55 | 32.26 | 9.20 | -1.59 | 9.57 | -3.79 | 11.78 | 32.43 | 13.47 | |
| 112 | 2 | 21.34 | 3.23 | -21.20 | 5.55 | 32.26 | 9.20 | -1.59 | 9.57 | -3.79 | 11.78 | 32.43 | 13.47 | |
| 113 | 3 | 55.80 | 23.75 | 0.23 | -6.16 | 20.91 | 27.86 | 56.16 | -3.03 | -11.48 | 8.15 | 31.48 | 28.13 | |
| 114 | 3 | 55.80 | 23.75 | 0.23 | -6.16 | 20.91 | 27.86 | 56.16 | -3.03 | -11.48 | 8.15 | 31.48 | 28.13 | |
| 115 | 3 | 54.95 | 23.75 | 0.23 | -5.98 | 20.91 | 27.60 | 54.92 | -3.03 | -11.48 | 8.31 | 31.48 | 27.69 | |
| 116 | 3 | 55.63 | 23.75 | 0.23 | -6.32 | 20.91 | 27.79 | 56.00 | -3.03 | -11.48 | 7.77 | 31.48 | 28.03 | |
| 117 | 3 | 54.85 | 23.75 | 0.23 | -6.11 | 20.91 | 27.55 | 54.77 | -3.03 | -11.48 | 8.87 | 31.48 | 27.70 | |
| 118 | 3 | 54.85 | 23.75 | 0.23 | -6.11 | 20.91 | 27.55 | 54.77 | -3.03 | -11.48 | 8.87 | 31.48 | 27.70 | |
| 119 | 3 | 50.13 | 23.75 | 0.23 | -5.47 | 20.91 | 26.05 | 47.15 | -3.03 | -11.48 | 8.21 | 31.48 | 24.76 | |
| 120 | 3 | 50.13 | 23.75 | 0.23 | -5.47 | 20.91 | 26.05 | 47.15 | -3.03 | -11.48 | 8.21 | 31.48 | 24.76 | |
| 121 | 3 | 51.16 | 9.19 | -16.90 | -4.95 | 6.34 | 21.02 | 42.65 | 1.42 | -8.95 | -9.64 | 21.14 | 19.13 | |
| 122 | 3 | 51.16 | 9.19 | -16.90 | -4.95 | 6.34 | 21.02 | 42.65 | 1.42 | -8.95 | -9.64 | 21.14 | 19.13 | |
| 123 | 3 | 51.15 | 9.19 | -16.90 | -6.02 | 6.34 | 20.87 | 42.91 | 1.42 | -8.95 | -9.80 | 21.14 | 19.21 | |
| 124 | 3 | 51.15 | 9.19 | -16.90 | -6.02 | 6.34 | 20.87 | 42.91 | 1.42 | -8.95 | -9.80 | 21.14 | 19.21 | |
| 125 | 3 | 51.18 | 9.19 | -16.90 | -5.94 | 6.34 | 20.89 | 42.96 | 1.42 | -8.95 | -9.41 | 21.14 | 19.28 | |
| 126 | 3 | 42.20 | 12.44 | -25.71 | 6.31 | 21.21 | 15.15 | 27.96 | -2.36 | -2.02 | -5.74 | 10.27 | 9.34 | |
| 127 | 3 | 41.86 | 12.44 | -25.71 | 6.48 | 21.21 | 15.06 | 27.03 | -2.36 | -2.02 | -4.24 | 10.27 | 9.28 | |
| 128 | 3 | 41.86 | 12.44 | -25.71 | 6.48 | 21.21 | 15.06 | 27.03 | -2.36 | -2.02 | -4.24 | 10.27 | 9.28 | |
| 129 | 3 | 41.86 | 12.44 | -25.71 | 6.48 | 21.21 | 15.06 | 27.03 | -2.36 | -2.02 | -4.24 | 10.27 | 9.28 | |
| 130 | 3 | 41.86 | 12.44 | -25.71 | 6.48 | 21.21 | 15.06 | 27.03 | -2.36 | -2.02 | -4.24 | 10.27 | 9.28 | |
| 131 | 3 | 41.68 | 12.44 | -25.71 | 5.75 | 21.21 | 14.92 | 26.34 | -2.36 | -2.02 | -3.25 | 10.27 | 9.22 | |
| 132 | 3 | 40.47 | 12.44 | -25.71 | 4.67 | 21.47 | 14.45 | 24.52 | -2.36 | -2.02 | -5.48 | 10.27 | 8.24 | |
| 133 | 3 | 40.47 | 12.44 | -25.71 | 4.67 | 21.47 | 14.45 | 24.52 | -2.36 | -2.02 | -5.48 | 10.27 | 8.24 | |
| 134 | 3 | 37.92 | 13.33 | 0.54 | 10.05 | 5.73 | 17.58 | 20.41 | 2.84 | -11.53 | 13.46 | 11.14 | 9.45 | |
| 135 | 3 | 19.44 | 2.54 | 17.16 | -3.07 | 11.76 | 9.57 | 13.42 | 6.85 | 2.32 | 4.74 | 21.65 | 11.77 | |
| 136 | 3 | 14.70 | -1.98 | 19.72 | -0.30 | 23.52 | 13.20 | 11.69 | 5.83 | 5.28 | 11.88 | 28.59 | 15.31 | |
| 137 | 3 | 12.95 | -1.98 | 19.72 | 0.48 | 23.52 | 13.03 | 10.50 | 5.83 | 5.28 | 14.34 | 28.59 | 15.52 | |
| 138 | 2 | 0.97 | -1.98 | 19.72 | -3.60 | 23.52 | 10.36 | 0.97 | 5.83 | 5.28 | 13.48 | 28.59 | 13.79 | |
| 139 | 2 | 0.29 | -1.98 | 19.72 | 7.35 | 23.52 | 12.07 | 0.15 | 5.83 | 5.28 | 14.88 | 28.59 | 13.89 | |
| 140 | 1 | -0.97 | -1.98 | 19.72 | 5.63 | 23.52 | 11.57 | -0.53 | 5.83 | 5.28 | 25.01 | 28.59 | 16.83 | |
| 141 | 1 | -0.86 | -1.98 | 19.72 | 7.33 | 23.52 | 11.88 | -0.44 | 5.83 | 5.28 | 27.09 | 28.59 | 17.43 | |
| 142 | 2 | 0.64 | -1.98 | 19.72 | 6.84 | 23.52 | 12.04 | 0.47 | 5.83 | 5.28 | 19.20 | 28.59 | 14.66 | |
| 143 | 1 | -0.85 | -1.98 | 19.72 | 6.67 | 23.52 | 11.77 | -0.52 | 5.83 | 5.28 | 13.01 | 28.59 | 13.46 | |
| 144 | 1 | -0.88 | -1.98 | 19.72 | 11.38 | 23.52 | 12.55 | -0.56 | 5.83 | 5.28 | 8.94 | 28.59 | 12.78 | |
| 145 | 1 | -0.92 | -1.98 | 19.72 | 11.76 | 23.52 | 12.60 | -0.56 | 5.83 | 5.28 | 2.29 | 28.59 | 11.67 | |
| 146 | 1 | -0.93 | -1.98 | 19.72 | -3.85 | 23.52 | 10.00 | -0.58 | 5.83 | 5.28 | 9.57 | 28.59 | 12.88 | |
| 147 | 1 | -0.89 | -1.98 | 19.72 | 8.06 | 23.52 | 11.99 | -0.47 | 5.83 | 5.28 | 27.98 | 28.59 | 17.68 | |
| 148 | 1 | -0.91 | -1.98 | 19.72 | 9.70 | 23.52 | 12.26 | -0.52 | 5.83 | 5.28 | -1.07 | 28.59 | 11.12 | |
| 149 | 1 | -0.89 | -1.98 | 19.72 | 5.71 | 23.52 | 11.60 | -0.47 | 5.83 | 5.28 | 26.25 | 28.59 | 17.19 | |
| 150 | 3 | 20.05 | 2.54 | 17.16 | -0.76 | 11.76 | 11.80 | 13.60 | 6.85 | 2.32 | 3.15 | 21.65 | 11.54 | |
| 151 | 1 | -0.26 | 7.04 | -3.95 | -1.33 | 7.97 | 1.89 | 0.31 | 7.64 | 25.04 | 19.41 | 25.73 | 18.41 | |
| 152 | 2 | 3.66 | 7.04 | -3.95 | 1.52 | 7.97 | 3.25 | 5.32 | 7.64 | 19.85 | 16.59 | 25.73 | 16.81 | |
| 153 | 1 | -0.50 | 13.33 | 0.54 | -2.37 | 5.73 | 3.35 | -0.09 | 2.84 | -11.53 | 5.06 | 11.14 | 1.48 | |
| 154 | 2 | 15.58 | 2.88 | -6.69 | 14.86 | -6.07 | 4.11 | 11.73 | 3.68 | 8.73 | 10.69 | 9.14 | 8.79 | |
| 155 | 2 | 15.52 | -2.66 | 16.28 | 10.22 | 1.12 | 8.10 | 11.71 | 2.98 | 7.74 | 15.64 | 17.75 | 11.16 | |
| 156 | 2 | 16.12 | 2.88 | -6.69 | 14.11 | -6.07 | 4.07 | 12.16 | 3.68 | 13.78 | 8.86 | 9.14 | 9.52 | |
| 157 | 2 | 1.53 | 4.26 | -9.73 | -0.80 | 17.36 | 2.52 | 2.99 | 10.48 | -7.15 | 1.95 | 28.46 | 10.86 | |
| 158 | 2 | 1.39 | 4.26 | -9.73 | -5.88 | 17.36 | 1.48 | 2.75 | 10.48 | -7.15 | -2.95 | 28.46 | 10.01 | |
| 159 | 2 | 1.23 | 4.26 | -9.73 | -10.38 | 17.36 | 0.55 | 2.33 | 10.48 | -7.15 | 1.80 | 28.46 | 10.73 | |
| 160 | 2 | 1.01 | 4.26 | -9.73 | 3.21 | 17.36 | 3.22 | 1.93 | 10.48 | -7.15 | 1.19 | 28.46 | 10.56 | |
| 161 | 1 | -0.71 | 4.26 | -9.73 | 0.20 | 17.36 | 2.28 | 0.03 | 10.48 | -7.15 | 11.06 | 28.46 | 11.89 | |
| 162 | 1 | -0.20 | 4.26 | -9.73 | -6.11 | 17.36 | 1.12 | 0.65 | 10.48 | -7.15 | 1.02 | 28.46 | 10.32 | |
| 163 | 2 | 1.29 | 23.75 | 0.23 | 7.71 | 20.91 | 14.08 | 1.40 | -3.03 | -11.48 | 17.71 | 31.48 | 11.26 | |
| 164 | 2 | 1.01 | 23.75 | 0.23 | -8.23 | 20.91 | 11.76 | 0.98 | -3.03 | -11.48 | 6.58 | 31.48 | 9.34 | |
| 165 | 1 | -0.69 | 23.75 | 0.23 | -7.79 | 20.91 | 11.58 | -0.16 | -3.03 | -11.48 | -1.79 | 31.48 | 7.75 | |
| 166 | 1 | -0.69 | 23.75 | 0.23 | -7.79 | 20.91 | 11.58 | -0.16 | -3.03 | -11.48 | -1.79 | 31.48 | 7.75 | |
| 167 | 1 | -0.69 | 23.75 | 0.23 | -7.79 | 20.91 | 11.58 | -0.16 | -3.03 | -11.48 | -1.79 | 31.48 | 7.75 | |
| 168 | 2 | -0.57 | 5.15 | -16.03 | 8.71 | 19.38 | 3.33 | -0.47 | 6.76 | -4.37 | 5.75 | 30.76 | 11.53 | |
| 169 | 1 | -1.02 | 5.15 | -16.03 | 5.23 | 19.38 | 2.54 | -0.14 | 6.76 | -4.37 | 13.59 | 30.76 | 12.89 | |
| 170 | 1 | -1.02 | 5.15 | -16.03 | 5.23 | 19.38 | 2.54 | -0.14 | 6.76 | -4.37 | 13.59 | 30.76 | 12.89 | |
| 171 | 2 | 20.93 | 3.23 | -21.20 | 3.37 | 32.26 | 8.82 | -1.38 | 9.57 | -3.79 | 9.25 | 32.43 | 13.09 | |
| 172 | 1 | -1.02 | 5.15 | -16.03 | 2.17 | 19.38 | 1.93 | -0.16 | 6.76 | -4.37 | 2.89 | 30.76 | 11.11 | |
| 173 | 1 | -1.02 | 5.15 | -16.03 | 2.17 | 19.38 | 1.93 | -0.16 | 6.76 | -4.37 | 2.89 | 30.76 | 11.11 | |
| 174 | 1 | -1.02 | 5.15 | -16.03 | 2.17 | 19.38 | 1.93 | -0.16 | 6.76 | -4.37 | 2.89 | 30.76 | 11.11 | |
| 175 | 1 | -1.06 | 5.15 | -16.03 | -2.04 | 19.38 | 1.08 | -0.26 | 6.76 | -4.37 | 12.65 | 30.76 | 12.71 | |
| 176 | 1 | -1.04 | 5.15 | -16.03 | 14.28 | 19.38 | 4.35 | -0.21 | 6.76 | -4.37 | 24.28 | 30.76 | 16.04 | |
| 177 | 1 | -1.06 | 5.15 | -16.03 | 9.75 | 19.38 | 3.44 | -0.17 | 6.76 | -4.37 | 10.21 | 30.76 | 12.32 | |
| 178 | 1 | -0.94 | 5.15 | -16.03 | 13.85 | 19.38 | 4.28 | -0.10 | 6.76 | -4.37 | 18.83 | 30.76 | 13.77 | |
| 179 | 1 | -1.05 | 5.15 | -16.03 | 13.25 | 19.38 | 4.14 | -0.21 | 6.76 | -4.37 | 22.57 | 30.76 | 15.55 | |
| 180 | 2 | -0.69 | 12.22 | -18.91 | 0.16 | 37.76 | 11.38 | -0.18 | 0.58 | -15.17 | 12.26 | 33.09 | 10.61 | |
| 181 | 2 | -0.68 | 12.22 | -18.91 | -12.72 | 37.76 | 9.24 | -0.18 | 0.58 | -15.17 | 0.86 | 33.09 | 8.71 | |
| 182 | 1 | -0.86 | 12.22 | -18.91 | 10.18 | 37.76 | 13.02 | -0.19 | 0.58 | -15.17 | 5.03 | 33.09 | 9.40 | |
| 183 | 1 | -0.59 | 11.58 | -17.34 | -2.34 | 34.66 | 10.11 | -0.18 | -8.09 | -13.36 | -0.02 | 30.10 | 6.42 | |
| 184 | 1 | -0.70 | 11.58 | -17.34 | 1.12 | 34.66 | 10.67 | -0.18 | -8.09 | -13.36 | 8.66 | 30.10 | 7.87 | |
| 185 | 2 | 0.57 | 2.03 | -3.33 | -1.59 | 35.15 | 11.33 | -0.95 | 12.59 | -1.02 | -4.81 | 27.95 | 10.29 | |
| 186 | 2 | 0.57 | 2.03 | -3.33 | -3.05 | 35.15 | 11.09 | -0.92 | 12.59 | -1.02 | 1.11 | 27.95 | 11.28 | |
| 187 | 2 | 0.46 | 2.03 | -3.33 | -5.28 | 35.15 | 10.70 | -0.92 | 12.59 | -1.02 | 2.72 | 27.95 | 11.55 | |
| 188 | 2 | 9.43 | 7.50 | 4.02 | -7.58 | 30.45 | 12.38 | 3.15 | 9.40 | 8.96 | 0.93 | 22.72 | 11.31 | |
| 189 | 2 | 11.58 | 7.50 | 4.02 | -8.33 | 30.45 | 12.61 | 3.83 | 9.40 | 8.96 | 1.89 | 22.72 | 11.59 | |
| 190 | 2 | 4.80 | 7.50 | 4.02 | -4.83 | 30.45 | 12.07 | 1.28 | 9.40 | 8.96 | 1.12 | 22.72 | 11.03 | |
| 191 | 1 | -1.00 | 2.03 | -3.33 | -3.12 | 35.15 | 10.81 | -0.32 | 12.59 | -1.02 | 7.47 | 27.95 | 12.44 | |
| 192 | 1 | -0.80 | 2.03 | -3.33 | 20.41 | 35.15 | 15.57 | -0.22 | 12.59 | -1.02 | -2.29 | 27.95 | 10.83 | |
| 193 | 1 | -0.79 | 18.23 | -13.10 | -15.20 | 33.82 | 9.46 | -0.16 | -8.93 | -8.93 | 11.82 | 33.55 | 10.15 | |
| 194 | 1 | -0.78 | 18.23 | -13.10 | -15.51 | 33.82 | 9.41 | -0.15 | -8.93 | -8.93 | 11.80 | 33.55 | 10.15 | |
| 195 | 1 | -0.37 | 7.50 | 4.02 | 2.26 | 30.45 | 12.38 | -0.54 | 9.40 | 8.96 | -3.70 | 22.72 | 9.93 | |
| 196 | 2 | 1.04 | 7.50 | 4.02 | 0.72 | 30.45 | 12.36 | -0.76 | 9.40 | 8.96 | 0.11 | 22.72 | 10.53 | |
| 197 | 1 | 0.48 | 7.50 | 4.02 | -2.52 | 30.45 | 11.73 | -0.55 | 9.40 | 8.96 | -1.59 | 22.72 | 10.28 | |
| 198 | 1 | -0.10 | 7.50 | 4.02 | -4.05 | 30.45 | 11.38 | -0.47 | 9.40 | 8.96 | 5.20 | 22.72 | 11.42 | |
| 199 | 1 | -0.10 | 7.50 | 4.02 | -4.05 | 30.45 | 11.38 | -0.47 | 9.40 | 8.96 | 5.20 | 22.72 | 11.42 | |
| 200 | 1 | -0.10 | 7.50 | 4.02 | -4.05 | 30.45 | 11.38 | -0.47 | 9.40 | 8.96 | 5.20 | 22.72 | 11.42 | |
| 201 | 2 | 1.06 | 7.50 | 4.02 | 1.72 | 30.45 | 12.53 | -0.69 | 9.40 | 8.96 | 0.91 | 22.72 | 10.67 | |
| 202 | 2 | 4.31 | 7.50 | 4.02 | -0.85 | 30.45 | 12.65 | 1.05 | 9.40 | 8.96 | 2.65 | 22.72 | 11.25 | |
| 203 | 2 | 1.67 | 7.50 | 4.02 | -1.30 | 30.45 | 12.13 | -0.73 | 9.40 | 8.96 | 4.61 | 22.72 | 11.28 | |
| 204 | 1 | 1.01 | 7.50 | 4.02 | -1.97 | 30.45 | 11.91 | -0.66 | 9.40 | 8.96 | 2.00 | 22.72 | 10.86 | |
| 205 | 3 | 68.13 | 23.48 | -12.64 | -6.65 | 33.82 | 36.78 | 50.81 | -1.29 | -9.01 | 0.09 | 25.85 | 24.24 | |
| 206 | 3 | 68.13 | 23.48 | -12.64 | -6.90 | 33.82 | 36.76 | 50.80 | -1.29 | -9.01 | -0.04 | 25.85 | 24.22 | |
| 207 | 3 | 66.96 | 18.23 | -13.10 | -8.02 | 33.82 | 36.95 | 53.71 | -8.93 | -8.93 | 0.71 | 33.55 | 26.38 | |
| 208 | 3 | 69.21 | 23.48 | -12.64 | -6.67 | 33.82 | 37.21 | 53.01 | -1.29 | -9.01 | 0.16 | 25.85 | 25.07 | |
| 209 | 3 | 68.37 | 18.23 | -13.10 | -7.67 | 33.82 | 37.62 | 53.68 | -8.93 | -8.93 | 0.24 | 33.55 | 26.31 | |
| 210 | 1 | -0.86 | 13.33 | 0.54 | 1.88 | 5.73 | 4.12 | -0.32 | 2.84 | -11.53 | 5.54 | 11.14 | 1.53 | |
| 211 | 2 | 3.08 | -2.66 | 16.28 | 10.92 | 1.12 | 5.75 | 8.15 | 2.98 | 0.62 | 13.81 | 17.75 | 8.66 | |
| 212 | 2 | 9.68 | -2.66 | 16.28 | 6.98 | 1.12 | 6.28 | 8.45 | 2.98 | -5.25 | 12.64 | 17.75 | 7.31 | |
| 213 | 1 | 0.64 | 4.26 | -9.73 | 0.65 | 17.36 | 2.64 | 1.43 | 10.48 | -7.15 | 3.58 | 28.46 | 10.88 | |
| 214 | 2 | 1.54 | 4.26 | -9.73 | 1.71 | 17.36 | 3.03 | 3.00 | 10.48 | -7.15 | 3.88 | 28.46 | 11.19 | |
| 215 | 1 | -0.82 | 23.75 | 0.23 | 10.66 | 20.91 | 14.20 | -0.17 | -3.03 | -11.48 | 8.86 | 31.48 | 9.52 | |
| 216 | 1 | -1.01 | 5.15 | -16.03 | 16.76 | 19.38 | 4.85 | -0.23 | 6.76 | -4.37 | 13.92 | 30.76 | 12.93 | |
| 217 | 1 | -0.39 | 11.58 | -17.34 | -8.70 | 34.66 | 9.08 | -0.10 | -8.09 | -13.36 | -14.69 | 30.10 | 3.99 | |
| 218 | 1 | 0.54 | 7.50 | 4.02 | -2.55 | 30.45 | 11.74 | -0.55 | 9.40 | 8.96 | 3.50 | 22.72 | 11.12 | |
| 219 | 1 | 0.47 | 7.50 | 4.02 | -2.22 | 30.45 | 11.78 | -0.58 | 9.40 | 8.96 | -1.42 | 22.72 | 10.30 | |
| 220 | 1 | 0.35 | 7.50 | 4.02 | 2.69 | 30.45 | 12.58 | -0.59 | 9.40 | 8.96 | 2.19 | 22.72 | 10.90 | |
| 221 | 2 | 1.05 | 7.50 | 4.02 | -0.62 | 30.45 | 12.14 | -0.73 | 9.40 | 8.96 | -4.28 | 22.72 | 9.80 | |
| 222 | 2 | 1.40 | 7.50 | 4.02 | -13.06 | 30.45 | 10.13 | -0.74 | 9.40 | 8.96 | 1.60 | 22.72 | 10.78 | |
| 223 | 1 | 0.56 | 7.50 | 4.02 | -3.11 | 30.45 | 11.65 | -0.59 | 9.40 | 8.96 | 3.58 | 22.72 | 11.13 | |

**Table S3.** Mean species assemblage responses (SARs) to five IHA metrics and overall species assemblage responses (OSARs) according to the weighted mean aggregation method in two projected periods of Horizons 2050 and 2090 in the Treene river catchment.

| Site | Stream order | Horizon 2050 | | | | | | Horizon 2090 | | | | | | |
| --- | --- | --- | --- | --- | --- | --- | --- | --- | --- | --- | --- | --- | --- | --- |
|  |  | Duration | Frequency | Magnitude | Rate | Timing | Weighted  mean | | Duration | Frequency | Magnitude | Rate | Timing | Weighted  mean |
| 1 | 2 | -3.14 | 8.74 | 0.16 | 18.27 | -6.26 | 3.56 | | -0.11 | 5.33 | 18.31 | -1.38 | -3.15 | 3.80 |
| 2 | 2 | -2.85 | 9.87 | 4.49 | 33.85 | 0.00 | 13.20 | | -0.75 | 12.55 | 16.16 | 24.61 | 3.43 | 13.43 |
| 3 | 2 | -2.85 | 9.87 | 4.49 | 33.85 | 0.00 | 13.20 | | -0.75 | 12.55 | 16.16 | 24.61 | 3.43 | 13.43 |
| 4 | 1 | -0.97 | 4.60 | 2.76 | 35.83 | -16.74 | 10.22 | | -0.26 | -10.51 | 0.47 | 36.16 | -8.19 | 8.97 |
| 5 | 1 | -0.72 | 2.09 | -0.41 | -2.05 | -6.71 | -1.56 | | -0.10 | 12.96 | 19.05 | 11.76 | -0.17 | 8.70 |
| 6 | 1 | -0.72 | 2.09 | -0.41 | -2.05 | -6.71 | -1.56 | | -0.10 | 12.96 | 19.05 | 11.76 | -0.17 | 8.70 |
| 7 | 1 | -0.72 | 2.09 | -0.41 | -2.05 | -6.71 | -1.56 | | -0.10 | 12.96 | 19.05 | 11.76 | -0.17 | 8.70 |
| 8 | 2 | -5.17 | 12.83 | -2.19 | 40.76 | -12.61 | 16.45 | | -1.55 | 15.97 | 8.38 | 39.27 | -5.31 | 16.00 |
| 9 | 2 | -5.17 | 12.83 | -2.19 | 40.76 | -12.61 | 16.45 | | -1.55 | 15.97 | 8.38 | 39.27 | -5.31 | 16.00 |
| 10 | 2 | -5.17 | 12.83 | -2.19 | 40.76 | -12.61 | 16.45 | | -1.55 | 15.97 | 8.38 | 39.27 | -5.31 | 16.00 |
| 11 | 2 | -4.41 | 12.83 | -2.19 | 40.76 | -12.61 | 16.56 | | -1.44 | 15.97 | 8.38 | 39.27 | -5.31 | 16.02 |
| 12 | 2 | -4.41 | 12.83 | -2.19 | 40.76 | -12.61 | 16.56 | | -1.44 | 15.97 | 8.38 | 39.27 | -5.31 | 16.02 |
| 13 | 2 | -4.41 | 12.83 | -2.19 | 40.76 | -12.61 | 16.56 | | -1.44 | 15.97 | 8.38 | 39.27 | -5.31 | 16.02 |
| 14 | 2 | -3.19 | 12.83 | -2.19 | 40.76 | -12.61 | 16.73 | | -1.05 | 15.97 | 8.38 | 39.27 | -5.31 | 16.09 |
| 15 | 2 | -3.19 | 12.83 | -2.19 | 40.76 | -12.61 | 16.73 | | -1.05 | 15.97 | 8.38 | 39.27 | -5.31 | 16.09 |
| 16 | 2 | -3.19 | 12.83 | -2.19 | 40.76 | -12.61 | 16.73 | | -1.05 | 15.97 | 8.38 | 39.27 | -5.31 | 16.09 |
| 17 | 3 | -3.27 | 3.53 | 2.08 | 13.28 | 3.93 | 3.91 | | -0.47 | -1.28 | 11.88 | 1.43 | -1.86 | 1.94 |
| 18 | 3 | -3.27 | 3.53 | 2.08 | 13.28 | 3.93 | 3.91 | | -0.47 | -1.28 | 11.88 | 1.43 | -1.86 | 1.94 |
| 19 | 3 | -3.27 | 3.53 | 2.08 | 13.28 | 3.93 | 3.91 | | -0.47 | -1.28 | 11.88 | 1.43 | -1.86 | 1.94 |
| 20 | 3 | 12.00 | 9.87 | 4.49 | 33.85 | 0.00 | 15.68 | | 8.56 | 12.55 | 16.16 | 24.61 | 3.43 | 14.99 |
| 21 | 3 | 12.00 | 9.87 | 4.49 | 33.85 | 0.00 | 15.68 | | 8.56 | 12.55 | 16.16 | 24.61 | 3.43 | 14.99 |
| 22 | 2 | -4.70 | 4.60 | 2.76 | 35.83 | -16.74 | 9.60 | | -1.21 | -10.51 | 0.47 | 36.16 | -8.19 | 8.81 |
| 23 | 1 | -0.85 | 4.60 | 2.76 | 35.83 | -16.74 | 10.24 | | -0.22 | -10.51 | 0.47 | 36.16 | -8.19 | 8.98 |
| 24 | 1 | -0.62 | 5.03 | -0.22 | 6.92 | 14.38 | 5.10 | | -0.14 | 2.41 | 11.33 | -8.68 | 13.44 | 3.67 |
| 25 | 2 | -5.74 | 7.25 | 4.27 | 1.38 | -3.39 | 0.75 | | -1.35 | -2.70 | 5.59 | -1.07 | -1.68 | -0.24 |
| 26 | 2 | -5.74 | 7.25 | 4.27 | 1.38 | -3.39 | 0.75 | | -1.35 | -2.70 | 5.59 | -1.07 | -1.68 | -0.24 |
| 27 | 2 | -5.74 | 7.25 | 4.27 | 1.38 | -3.39 | 0.75 | | -1.35 | -2.70 | 5.59 | -1.07 | -1.68 | -0.24 |
| 28 | 1 | -0.82 | 5.03 | -0.22 | 6.92 | 14.38 | 5.06 | | -0.18 | 2.41 | 11.33 | -8.68 | 13.44 | 3.67 |
| 29 | 1 | -0.63 | 8.74 | 0.16 | 18.27 | -6.26 | 4.06 | | -0.06 | 5.33 | 18.31 | -1.38 | -3.15 | 3.81 |
| 30 | 1 | -0.63 | 8.74 | 0.16 | 18.27 | -6.26 | 4.06 | | -0.06 | 5.33 | 18.31 | -1.38 | -3.15 | 3.81 |
| 31 | 2 | -1.90 | 8.74 | 0.16 | 18.27 | -6.26 | 3.80 | | -0.12 | 5.33 | 18.31 | -1.38 | -3.15 | 3.80 |
| 32 | 2 | -4.40 | 13.44 | 3.20 | 15.73 | 0.22 | 5.64 | | -1.02 | 7.69 | -2.51 | 16.31 | -5.70 | 2.96 |
| 33 | 2 | -4.40 | 13.44 | 3.20 | 15.73 | 0.22 | 5.64 | | -1.02 | 7.69 | -2.51 | 16.31 | -5.70 | 2.96 |
| 34 | 2 | -4.40 | 13.44 | 3.20 | 15.73 | 0.22 | 5.64 | | -1.02 | 7.69 | -2.51 | 16.31 | -5.70 | 2.96 |
| 35 | 2 | -4.40 | 13.44 | 3.20 | 15.73 | 0.22 | 5.64 | | -1.02 | 7.69 | -2.51 | 16.31 | -5.70 | 2.96 |
| 36 | 3 | 11.76 | 6.99 | 3.89 | 31.66 | 1.72 | 14.61 | | 8.50 | 14.36 | 14.77 | 15.80 | 5.29 | 11.75 |
| 37 | 3 | 11.76 | 6.99 | 3.89 | 31.66 | 1.72 | 14.61 | | 8.50 | 14.36 | 14.77 | 15.80 | 5.29 | 11.75 |
| 38 | 3 | 11.76 | 6.99 | 3.89 | 31.66 | 1.72 | 14.61 | | 8.50 | 14.36 | 14.77 | 15.80 | 5.29 | 11.75 |
| 39 | 3 | 11.76 | 6.99 | 3.89 | 31.66 | 1.72 | 14.61 | | 8.50 | 14.36 | 14.77 | 15.80 | 5.29 | 11.75 |
| 40 | 3 | -2.95 | 3.53 | 2.08 | 13.28 | 3.93 | 3.97 | | -0.53 | -1.28 | 11.88 | 1.43 | -1.86 | 1.93 |
| 41 | 3 | -2.95 | 3.53 | 2.08 | 13.28 | 3.93 | 3.97 | | -0.53 | -1.28 | 11.88 | 1.43 | -1.86 | 1.93 |
| 42 | 3 | -2.95 | 3.53 | 2.08 | 13.28 | 3.93 | 3.97 | | -0.53 | -1.28 | 11.88 | 1.43 | -1.86 | 1.93 |
| 43 | 3 | -2.95 | 3.53 | 2.08 | 13.28 | 3.93 | 3.97 | | -0.53 | -1.28 | 11.88 | 1.43 | -1.86 | 1.93 |
| 44 | 3 | -1.08 | 5.03 | -0.22 | 6.92 | 14.38 | 5.01 | | -0.91 | 2.41 | 11.33 | -8.68 | 13.44 | 3.52 |
| 45 | 3 | -1.08 | 5.03 | -0.22 | 6.92 | 14.38 | 5.01 | | -0.91 | 2.41 | 11.33 | -8.68 | 13.44 | 3.52 |
| 46 | 3 | -1.08 | 5.03 | -0.22 | 6.92 | 14.38 | 5.01 | | -0.91 | 2.41 | 11.33 | -8.68 | 13.44 | 3.52 |
| 47 | 3 | -1.08 | 5.03 | -0.22 | 6.92 | 14.38 | 5.01 | | -0.91 | 2.41 | 11.33 | -8.68 | 13.44 | 3.52 |
| 48 | 1 | -0.76 | 4.60 | 2.76 | 35.83 | -16.74 | 10.25 | | -0.20 | -10.51 | 0.47 | 36.16 | -8.19 | 8.98 |
| 49 | 1 | -0.76 | 4.60 | 2.76 | 35.83 | -16.74 | 10.25 | | -0.20 | -10.51 | 0.47 | 36.16 | -8.19 | 8.98 |
| 50 | 1 | -0.76 | 4.60 | 2.76 | 35.83 | -16.74 | 10.25 | | -0.20 | -10.51 | 0.47 | 36.16 | -8.19 | 8.98 |
| 51 | 1 | -0.64 | 18.90 | 2.75 | -4.72 | 15.71 | 6.40 | | -0.15 | 1.58 | 8.34 | 7.56 | 1.83 | 3.83 |
| 52 | 1 | -0.64 | 18.90 | 2.75 | -4.72 | 15.71 | 6.40 | | -0.15 | 1.58 | 8.34 | 7.56 | 1.83 | 3.83 |
| 53 | 1 | -0.69 | 5.03 | -0.22 | 6.92 | 14.38 | 5.08 | | -0.15 | 2.41 | 11.33 | -8.68 | 13.44 | 3.67 |
| 54 | 1 | -0.69 | 5.03 | -0.22 | 6.92 | 14.38 | 5.08 | | -0.15 | 2.41 | 11.33 | -8.68 | 13.44 | 3.67 |
| 55 | 3 | -4.85 | 18.90 | 2.75 | -4.72 | 15.71 | 5.56 | | -0.51 | 1.58 | 8.34 | 7.56 | 1.83 | 3.76 |
| 56 | 3 | -4.85 | 18.90 | 2.75 | -4.72 | 15.71 | 5.56 | | -0.51 | 1.58 | 8.34 | 7.56 | 1.83 | 3.76 |
| 57 | 3 | -4.85 | 18.90 | 2.75 | -4.72 | 15.71 | 5.56 | | -0.51 | 1.58 | 8.34 | 7.56 | 1.83 | 3.76 |
| 58 | 3 | -4.85 | 18.90 | 2.75 | -4.72 | 15.71 | 5.56 | | -0.51 | 1.58 | 8.34 | 7.56 | 1.83 | 3.76 |
| 59 | 3 | -4.85 | 18.90 | 2.75 | -4.72 | 15.71 | 5.56 | | -0.51 | 1.58 | 8.34 | 7.56 | 1.83 | 3.76 |
| 60 | 3 | -4.85 | 18.90 | 2.75 | -4.72 | 15.71 | 5.56 | | -0.51 | 1.58 | 8.34 | 7.56 | 1.83 | 3.76 |
| 61 | 1 | -0.62 | 4.60 | 2.76 | 35.83 | -16.74 | 10.28 | | -0.17 | -10.51 | 0.47 | 36.16 | -8.19 | 8.99 |
| 62 | 1 | -1.28 | 13.44 | 3.20 | 15.73 | 0.22 | 6.26 | | -0.31 | 7.69 | -2.51 | 16.31 | -5.70 | 3.10 |
| 63 | 2 | -2.05 | 13.44 | 3.20 | 15.73 | 0.22 | 6.11 | | -0.49 | 7.69 | -2.51 | 16.31 | -5.70 | 3.06 |
| 64 | 2 | -2.98 | 13.44 | 3.20 | 15.73 | 0.22 | 5.92 | | -0.69 | 7.69 | -2.51 | 16.31 | -5.70 | 3.02 |
| 65 | 2 | -5.25 | 12.83 | -2.19 | 40.76 | -12.61 | 16.44 | | -1.50 | 15.97 | 8.38 | 39.27 | -5.31 | 16.01 |
| 66 | 3 | -3.20 | 3.53 | 2.08 | 13.28 | 3.93 | 3.92 | | -0.48 | -1.28 | 11.88 | 1.43 | -1.86 | 1.94 |
| 67 | 3 | -3.20 | 3.53 | 2.08 | 13.28 | 3.93 | 3.92 | | -0.48 | -1.28 | 11.88 | 1.43 | -1.86 | 1.94 |

## Figures


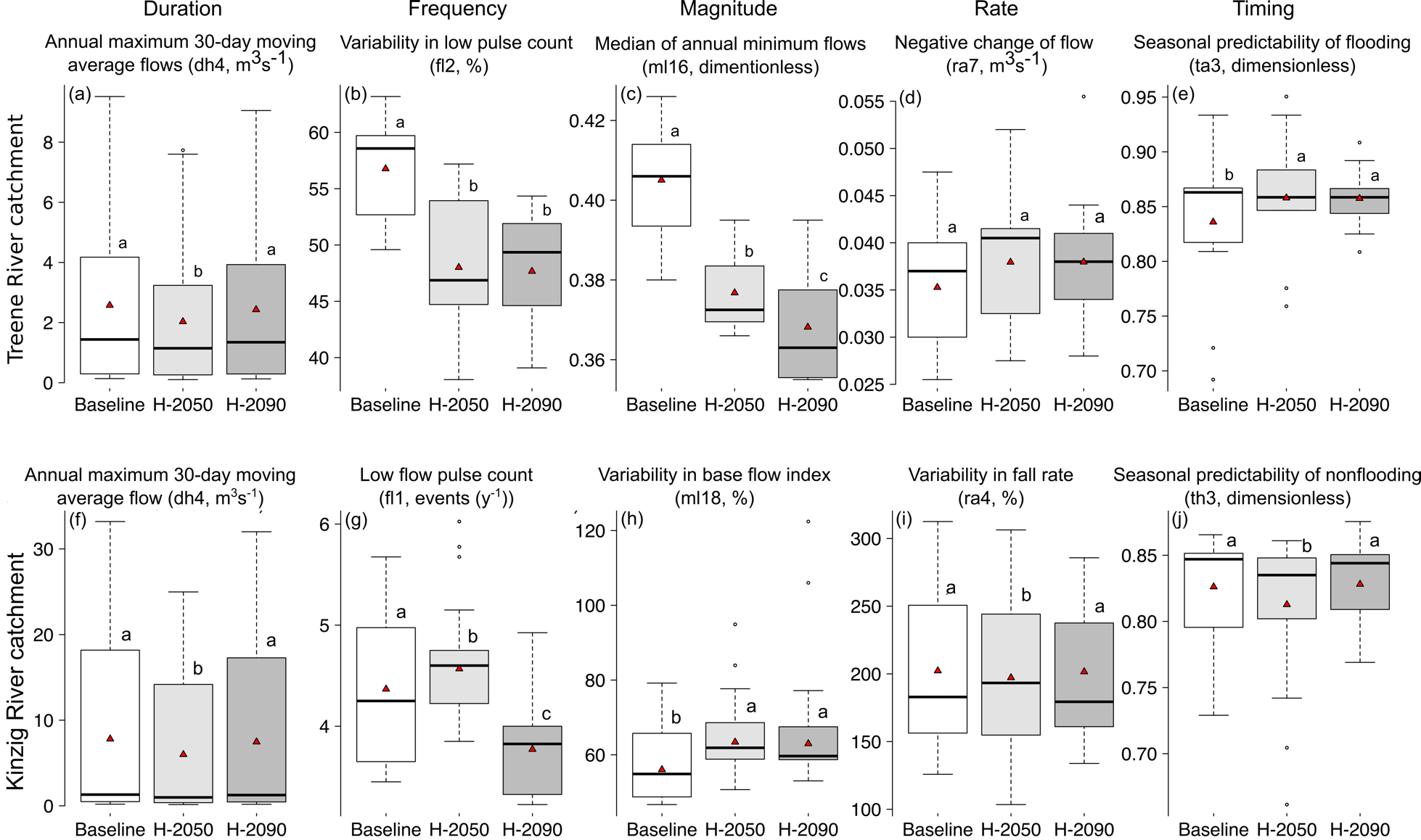


**Fig. S1** The boxplots (bar – median; red triangular – mean; box – 1^st^ and 3^rd^ interquartile range) show the potential changes in the IHA metrics at the sampling sites of the Treene (a-e) and Kinzig (f-j) catchments for the three defined 20-year periods of baseline (1998 – 2017), horizon 2050 (2046 – 2065) and horizon 2090 (2080 – 2099). The characters above each box shows whether the values would change significantly (p < 0.05; dissimilar characters) in the future or not (similar characters).


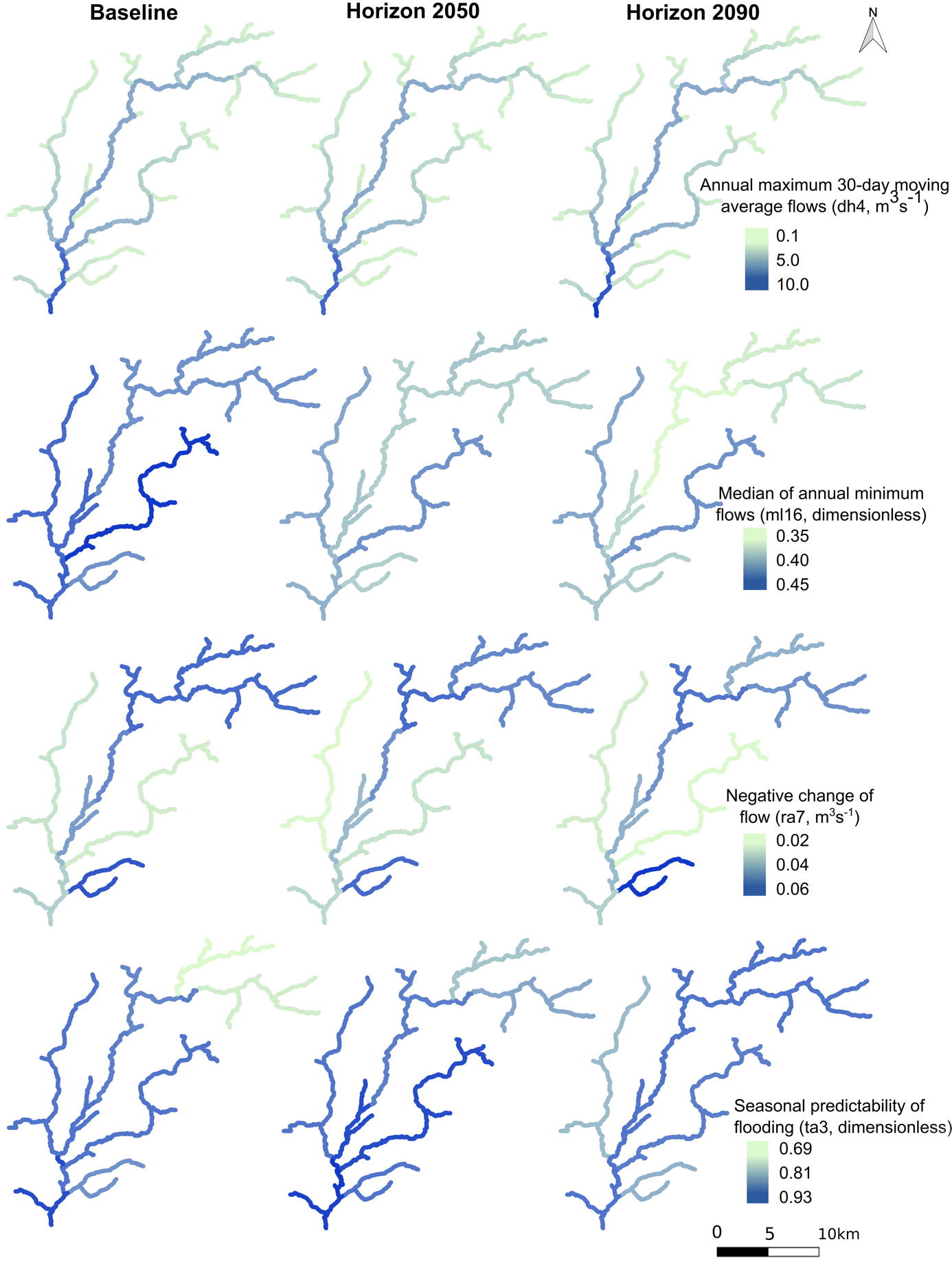


**Fig. S2** Potential changes in flow conditions according to each IHA metric, comparing the values during the baseline period with two projected periods in the Treene catchment (abbreviations in Table 2).

**
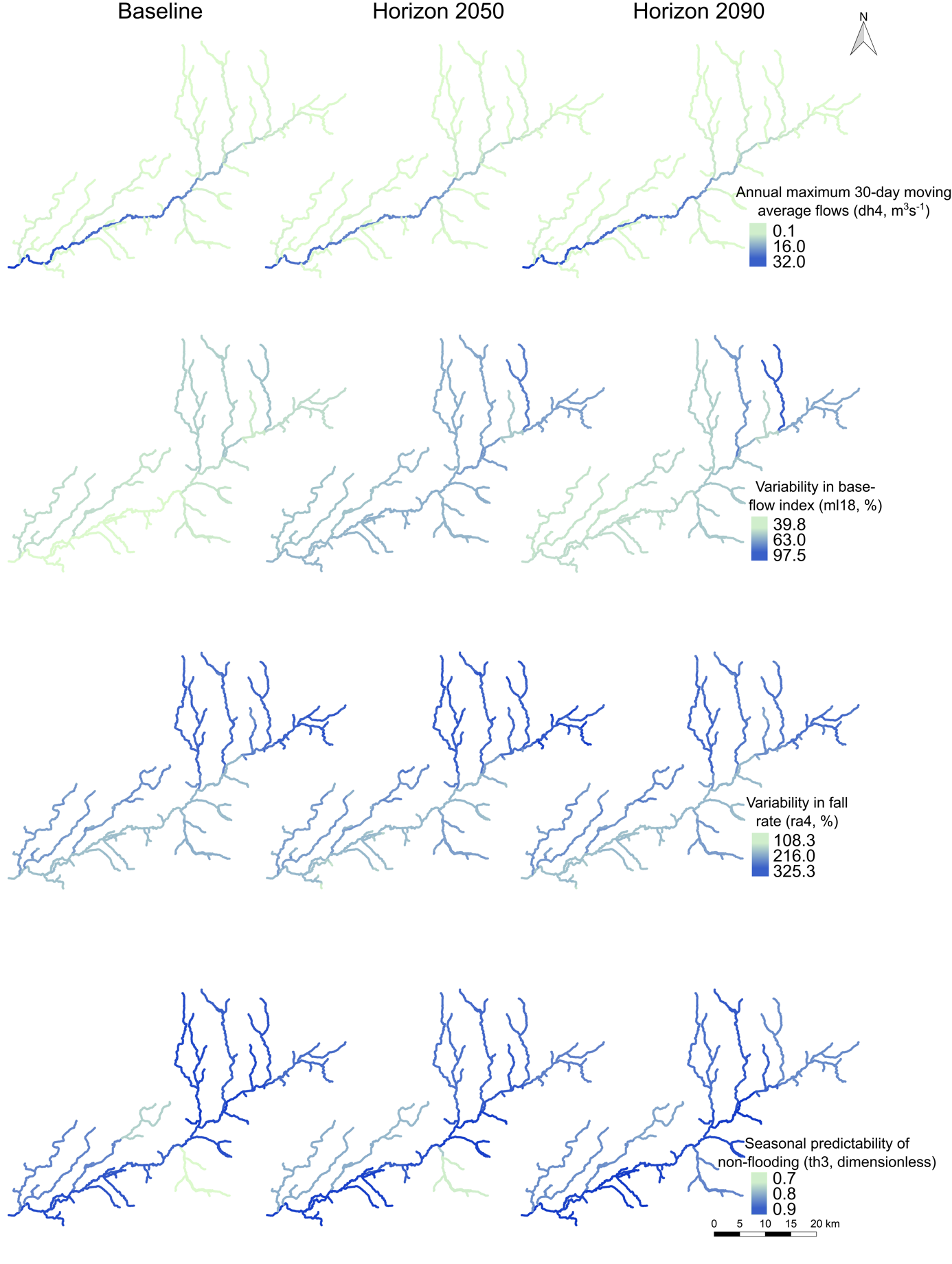
**

**Fig. S3** Potential changes in flow conditions according to each IHA metric, comparing the values during the baseline period with two projected periods in the Kinzig catchment (abbreviations in Table 2).


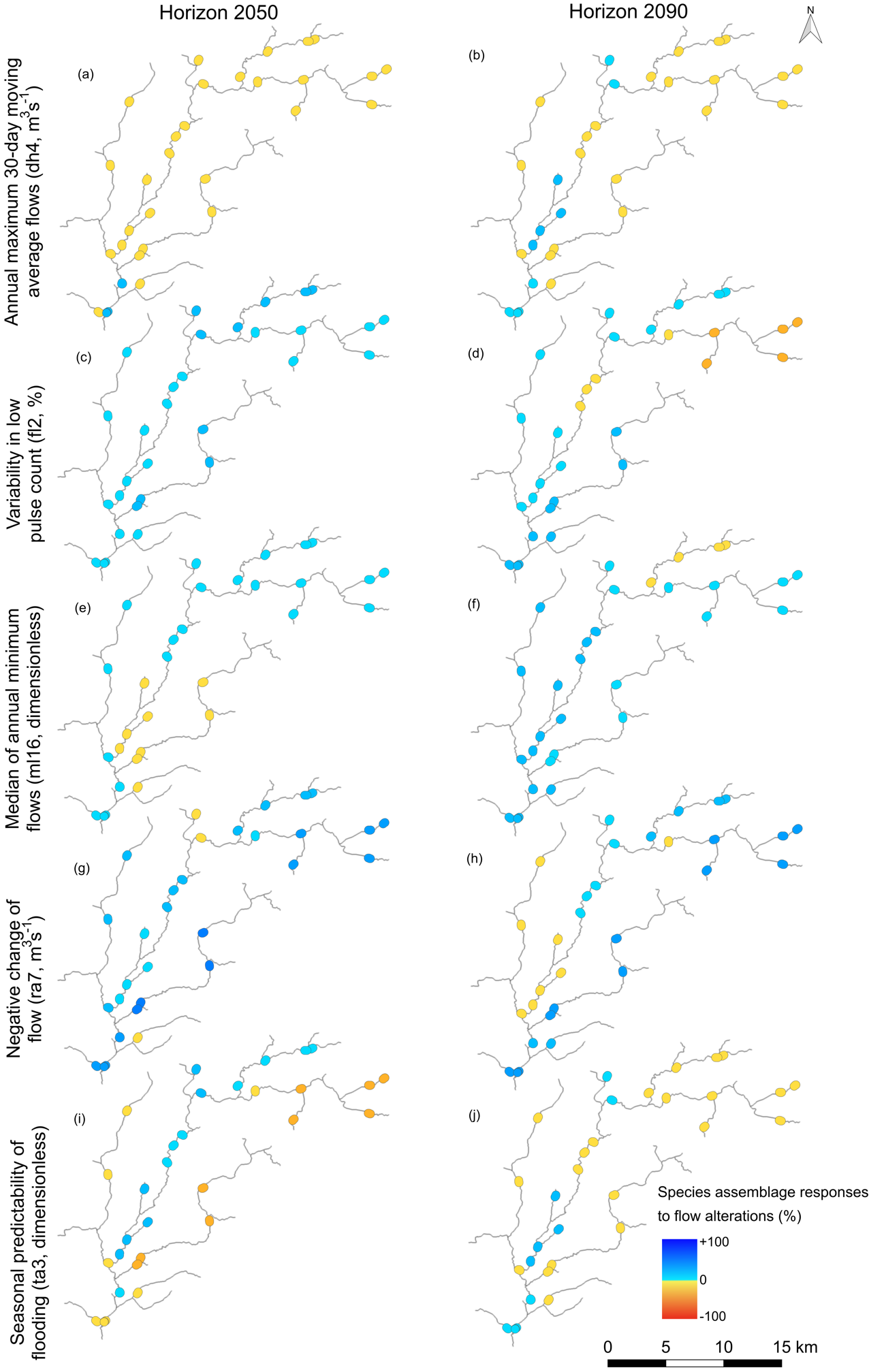


**Fig. S4** Potential response of species assemblages (SARs) in river reaches in horizons 2050 (left side) and 2090 (right side), according to changes in each IHA metric, in the Treene catchment.


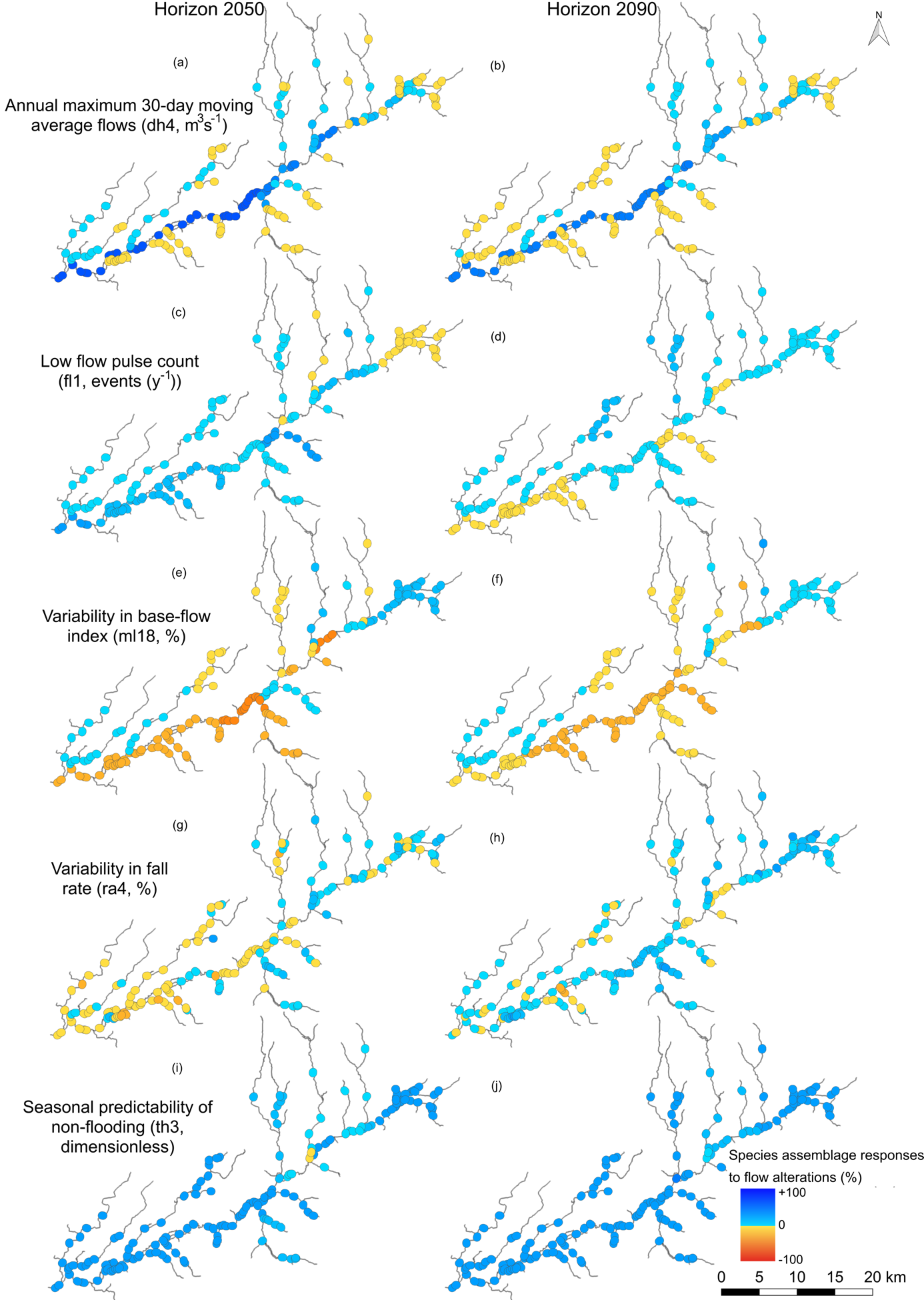


**Fig. S5** Potential response of species assemblages (SARs) in river reaches in horizons 2050 (left side) and 2090 (right side), according to changes in each IHA metric, in the Kinzig catchment.


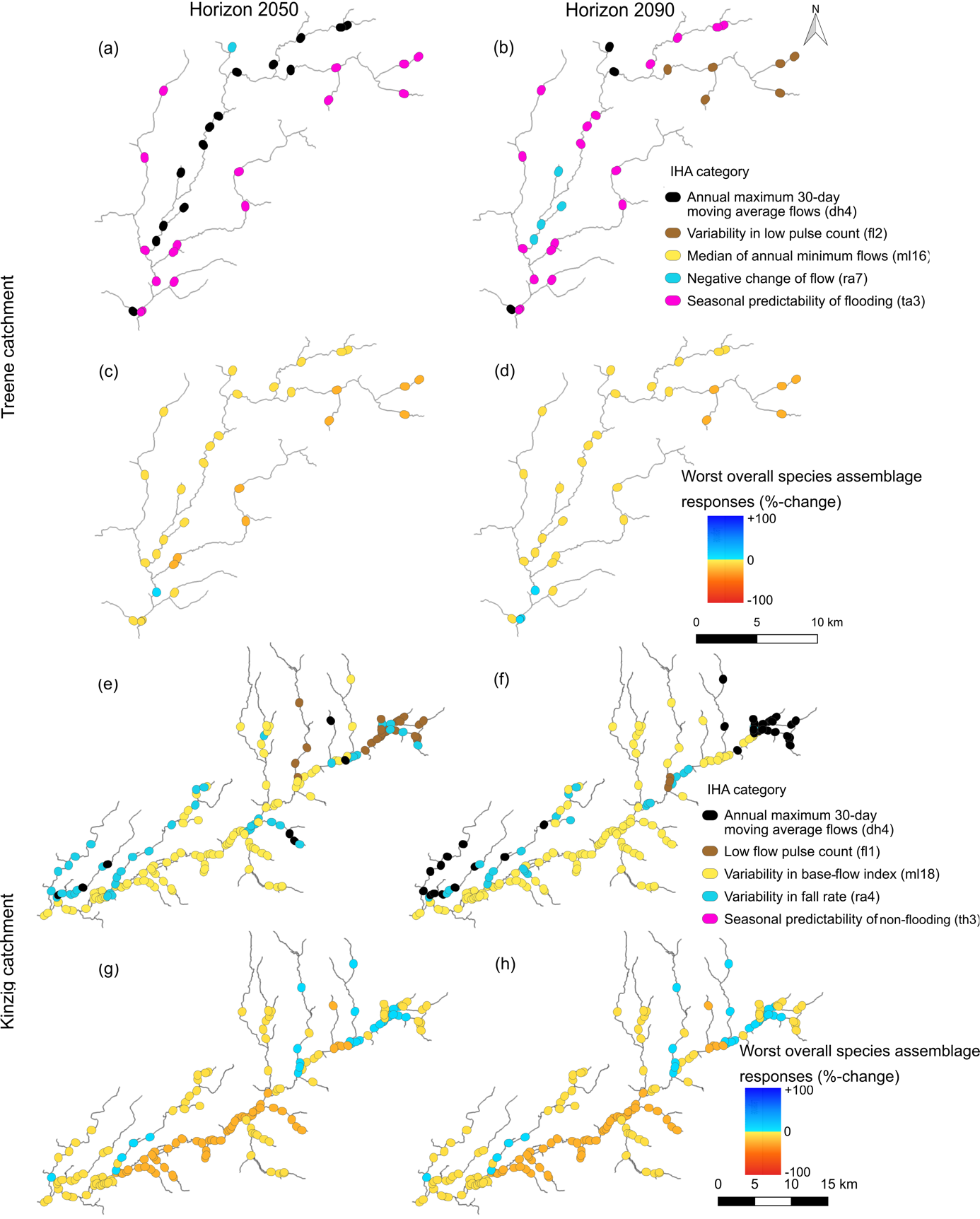


**Fig. S6** The worst overall species assemblage response (WOCR, eq. 5) in each river reach (c, d, g and h) according to the metric with the worst impact (worst scenario) on macroinvertebrates (a, b, e and f). All sub-figures on the left side (a, c, e and g) show the results for horizon 2050, while sub-figures on the right side (b, d, f and h) show the results for horizon 2090.
